# Monomeric C‐reactive protein via endothelial CD31 for neurovascular inflammation in an ApoE genotype‐dependent pattern: A risk factor for Alzheimer’s disease?

**DOI:** 10.1111/acel.13501

**Published:** 2021-10-23

**Authors:** Zhengrong Zhang, Hana Na, Qini Gan, Qiushan Tao, Yuriy Alekseyev, Junming Hu, Zili Yan, Jack B. Yang, Hua Tian, Shenyu Zhu, Qiang Li, Ibraheem M. Rajab, Jan Krizysztof Blusztajn, Benjamin Wolozin, Andrew Emili, Xiaoling Zhang, Thor Stein, Lawrence A. Potempa, Wei Qiao Qiu

**Affiliations:** ^1^ Department of Pharmacology and Experimental Therapeutics Boston University School of Medicine Boston Massachusetts USA; ^2^ Microarray and Sequencing Core Facility Boston University School of Medicine Boston Massachusetts USA; ^3^ Department of Medicine Boston University School of Medicine Boston Massachusetts USA; ^4^ Department of Pharmacology Xiaman Medical College Xiaman China; ^5^ Nursing School Qiqihar Medical University Qiqihar China; ^6^ Roosevelt University College of Pharmacy Schaumburg Illinois USA; ^7^ Department of Pathology and Laboratory Medicine Boston University School of Medicine Boston Massachusetts USA; ^8^ Department of Biochemistry Boston University School of Medicine Boston Massachusetts USA; ^9^ Alzheimer’s Disease Center Boston University School of Medicine Boston Massachusetts USA; ^10^ VA Boston Healthcare System Boston Massachusetts USA; ^11^ Department of Veterans Affairs Medical Center Bedford Massachusetts USA; ^12^ Department of Psychiatry Boston University School of Medicine Boston Massachusetts USA

**Keywords:** Alzheimer’s disease (AD), Apolipoprotein E, CD31, cerebrovascular, endothelia, lymphocyte extravasation, monomeric C‐reactive protein, neuroinflammation

## Abstract

In chronic peripheral inflammation, endothelia in brain capillary beds could play a role for the apolipoprotein E4 (ApoE4)‐mediated risk for Alzheimer's disease (AD) risk. Using human brain tissues, here we demonstrate that the interactions of endothelial CD31 with monomeric C‐reactive protein (mCRP) versus ApoE were linked with shortened neurovasculature for AD pathology and cognition. Using ApoE knock‐in mice, we discovered that intraperitoneal injection of mCRP, via binding to CD31 on endothelial surface and increased CD31 phosphorylation (pCD31), leading to cerebrovascular damage and the extravasation of T lymphocytes into the ApoE4 brain. While mCRP was bound to endothelial CD31 in a dose‐ and time‐dependent manner, knockdown of CD31 significantly decreased mCRP binding and altered the expressions of vascular‐inflammatory factors including vWF, NF‐κB and p‐eNOS. RNAseq revealed endothelial pathways related to oxidative phosphorylation and AD pathogenesis were enhanced, but endothelial pathways involving in epigenetics and vasculogenesis were inhibited in ApoE4. This is the first report providing some evidence on the ApoE4‐mCRP‐CD31 pathway for the cross talk between peripheral inflammation and cerebrovasculature leading to AD risk.

## Graphical abstract

Our study revealed a novel evidence that links ApoE genotype and cerebrovascular inflammation during peripheral chronic inflammation, which mirrors ApoE4 as a risk factor via endothelial CD31 for AD.

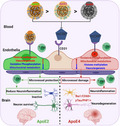

## 1 INTRODUCTION

An important feature of Alzheimer's disease (AD) is that the cerebrovasculature is altered in the brain (Iadecola & Gottesman, 2018). As peripheral chronic inflammation is associated with increase AD risk (Cao & Zheng, 2018), blood‐facing endothelia in the structure of capillary beds is first exposed to some blood proinflammatory factor(s) including C‐reactive protein (CRP) which might bind to a yet undetermined endothelial cell receptors to cause cerebrovascular neuroinflammation in AD.

Our human study found that elevated CRP is associated with an increased risk of AD in Apolipoprotein E4 (ApoE4) carriers but not in ApoE3 or ApoE2 carriers (Tao et al., 2018). It is shown that ApoE4 allele increases AD risk (Strittmatter et al., 1993) in part by disrupting the brain‐facing cells, pericytes and astrocytes, of the blood–brain barrier (BBB; Blanchard et al., 2020; Montagne et al., 2020). However, it is unclear whether ApoE4 also affects blood‐facing endothelia of the capillary beds. CRP plays a role in the immune response to toxins or injuries in peripheral inflammation (Stephensen & Gildengorin, 2000) with two forms in the body: (1) native pentameric CRP (pCRP) oligoprotein is produced during active inflammatory reactions (Slevin & Krupinski, 2009); and (2) monomeric CRP (mCRP) is produced during and after the acute phase by the irreversible dissociation of pCRP; these CRP monomers have a much lower aqueous solubility than pCRP and cause tissue damage (Caprio et al., 2018). There were two possibilities: (1) CRP is the key factor in peripheral inflammation exposing to endothelia and directly involved in ApoE4‐related AD pathogenesis; (2) CRP is a biomarker for peripheral chronic low‐grade inflammation, and other proinflammatory factors impact on ApoE4 to increase AD risk. Because CRP levels increase with age (Stephensen & Gildengorin, 2000; Tao et al., 2018) and mCRP plays a role in the pathogenesis of peripheral vascular diseases including cardiovascular diseases (Wang et al., 2015) and poststroke inflammation (Slevin et al., 2010), we hypothesized that mCRP plays some roles causing cerebrovascular pathology for the vascular risk of AD. Direct injection of mCRP into the hippocampus of 3xTg AD model mice enhanced the severity of AD‐like pathology in the brain (Slevin et al., 2015), but whether peripheral mCRP and ApoE isoforms act together on some receptor in brain endothelia and cause some brain pathology in non‐AD transgenic mice is unknown.

Taken together, these lines of evidence led us to hypothesize and test whether some endothelial surface protein mediates the actions of peripheral mCRP on cerebrovasculature in the AD brain in an ApoE genotype‐dependent pattern. The current study used human tissues and ApoE mouse models to characterize endothelial proteins and identified PECAM‐1 (CD31) as a receptor for peripheral mCRP to induce phosphorylation of CD31. We discovered that mCRP‐CD31 binding mediates differential endothelial responses depending on ApoE genotype, increasing cerebrovascular inflammation and some AD pathological features in ApoE4 animals. CD31 is a cellular adhesion and signaling receptor comprising six extracellular immunoglobulin‐like homology domains, a transmembrane domain and a cytoplasmic domain that becomes serine and tyrosine phosphorylated upon cellular activation to regulate vascular inflammation (Newton et al., 1997; Privratsky & Newman, 2014). It has been reported that CD31 mediates vascular‐immune regulation in peripheral system (Privratsky & Newman, 2014), yet its role in central nervous system is unclear.

## 2 METHODS

For all the quantification measurements described below, the researchers who directly conducted counting and other measurements were blinded to ApoE genotypes, treatments and AD diagnosis.

### 2.1 Human FHS ApoE and CRP data

Human serum CRP concentrations were obtained from the existing data of the Framingham Heart Study (FHS) offspring cohort (Gen 2). The source population comprised 3239 participants who met the following criteria: (1) were aged 30 years or older (range: 33–89 years, mean: 61.1 ± 9.5 years) at the time of the 7th health exam (1998–2001), and (2) consented to use their genetic information (i.e., ApoE genotype). Human ApoE genotype were divided into three groups: ApoE2 (ApoE2/2 or 2/3, *n* = 463), ApoE3 (ApoE3/3, *n* = 2076), and ApoE4 (ApoE3/4 or 4/4, *n* = 665). Those with ApoE2/4 (*n* = 62) were excluded in the analysis.

### 2.2 Human brain tissue and characterization

Brain temporal lobe tissues from 8 cognitive controls and 10 AD patients were obtained from the Brain Bank of the Boston University Alzheimer's Disease Center (BU ADC). The clinical features and ApoE genotypes are listed in Table S1. Immunostaining and Western blots as described above were used to characterize the levels of mCRP, the binding of mCRP‐CD31 or ApoE‐CD31, and the levels of pCD31 in the brain tissues. The lengths of CD31^+^ microvessel were measured from 3D imaging and the terminal ends of CD31^+^ microvessel were counted from 2D imaging. This process was conducted by researchers blinded to the diagnoses.

### 2.3 Mice and experimental treatments

Human ApoE genetic knock‐in mice and ApoE knockout mice were purchased from Taconic Biosciences, Inc. (APOE2: #1547‐F, APOE3: #1548‐F, APOE4: #1549‐F, ApoE^−/−^). The human ApoE gene (either the ApoE2, ApoE3 or ApoE4 allele) replaces the endogenous mouse ApoE gene in these mice. C57BL/6 wild‐type mice were purchased from Jackson Laboratory (#000664) to be used as a control group.

All mice were maintained in microisolator housing in the animal facility at Boston University School of Medicine. Recombinant mCRP was produced as described (Potempa et al., 2015). Female ApoE mice aged 9–11 months received an intraperitoneally (i.p.) injection with mCRP (200 µg/kg) in three days during 10am‐12pm per week (Monday, Wednesday and Friday) for 6 weeks. This dose was chosen based on previous pharmacokinetic studies (Nakayama et al., 1984; Tanigaki et al., 2013). mCRP was dissolved in phosphate‐buffered saline (PBS) before injection. Vehicle‐treated mice were injected with PBS only as a control (*n* = 15–18 mice in each examined condition). All animal procedures were performed in accordance with the National Institutes of Health Guide for the Care and Use of Laboratory Animals and were approved by the Boston University Animal Care and Use Committee.

### 2.4 Isolation, culture, and characterization of CD31^+^ brain endothelial cells (BECs)

Brain tissue samples including the cortex and hippocampus obtained from experimental mice that did or did not receive the mCRP treatment were gently dissociated into single‐cell suspensions using the Adult Brain Dissociation kit (#130107677, Miltenyi Biotec). Single‐cell isolation and characterization of CD31^+^ cells were performed as previously described with a minor modification (Yousef et al., 2018). Mice were deeply anesthetized by isoflurane and perfused by cold PBS. Brains were removed and dissected into the cortex and hippocampus using forceps in sterile conditions. The tissues were cut into eight slices with a razor blade and dissociated into a cell suspension using enzymatic buffer. Briefly, the tissues were enzymatically digested with the components for the mechanical dissociation step in the gentleMACS™ Octo Heat Dissociator. Following dissociation, myelin and cell debris were removed using the Debris Removal Solution. The procedure was followed by subsequent removal of erythrocytes using the Red Blood Cell Removal Solution. At this stage, we applied two methods: (1) flow cytometry to characterize the molecular signature of the cells; and (2) CD31 microbead‐based isolation and culture of BECs.

1. Flow cytometry: The cell pellet was resuspended in FACS buffer (0.5% BSA, 2 mM EDTA in PBS) and labeled with anti‐mouse CD31‐PE (1:50, #130‐111‐354, Miltenyi Biotec), anti‐mouse CD45‐FITC (1:50, #130‐116‐500, Miltenyi Biotec) and NIR for exclusion of dead cells (1:10^3^, #425301, BioLegend). Using flow cytometry (BD Bioscience), the cell samples in 0.5 ml FACS buffer were separated into different cell populations, and CD31^+^ BECs were sorted in bulk for further experiments.
2. Microbeads: Endothelial cells were enriched by depletion of CD45^+^ cells with CD45 microbeads (#130‐052‐301, Miltenyi Biotec) followed by positive selection using CD31 microbeads (#130‐097‐418, Miltenyi Biotec) in the magnetic separator. CD31^+^ BECs were resuspended in fresh EBM‐2 basal medium with all supplements (#CC‐3202, EGM™‐2‐MV BulletKit™, Lonza). Then, the BECs were seeded in 96‐well plates coated with collagen type I (5 µg/ml, #354231, BD Bioscience) at a density of 10^4^ cells per well. The medium was changed every 2 days.

On day 5, WT brain endothelial cells were treated in vitro with different concentrations of mCRP or vehicle control and incubated for different periods of time up to 24 hours (h). To explore the effects of different ApoE protein isoforms on mCRP, recombinant ApoE2, ApoE3 or ApoE4 (Perotech, Inc.) was added to CD31^+^ BECs at final concentrations of 0.03–3 μM and incubated for 1 h before mCRP was added at a final concentration of 10 μg/ml. The experimental cells were fixed and processed for ApoE or mCRP and CD31 colocalization analysis using the proximity ligation assay (PLA) approach. Cells were incubated with primary antibodies (anti‐phos‐CD31, anti‐CD31, and anti‐mCRP) and subsequently stained with secondary antibodies (Invitrogen).

### 2.5 Immunofluorescence characterization

Immunofluorescence was used to characterize the postmortem human‐ and mouse brains. Mouse brains were collected after PBS perfusion, post‐fixed in 4% paraformaldehyde for 48 h, and changed to 30% sucrose in PBS at 4°C. Coronal cryosections (30 μm in thickness) were used for the free‐floating staining method. For frozen human postmortem brain, the sample was embedded in OCT compound, cut into 16 µm thick cryosections and mounted on gelatin‐coated histological slides. The sections were allowed to air dry for 30 min and immediately fixed in ice‐cold fixation buffer for 15 min. Brain slides were preincubated in blocking solution with 5% [vol/vol] horse serum (Sigma‐Aldrich) in 1· Tris‐buffered saline for 2 h at room temperature. The slides were incubated individually with primary antibodies overnight. Brain slides were then stained with secondary antibodies conjugated with Alexa Fluor 488, 594, 549, or 647 (1:500, Thermo Fisher Scientific) for 1 h at room temperature. The sections were mounted with ProLong Gold antifade reagent with DAPI for nuclear staining (#P36935, Thermo Fisher Scientific). The stained slides were observed under fluorescence microscopy (Carl Zeiss).

The following primary antibodies were used: (1) 3H12 antibody (1:50) against mCRP (Ying et al., 1989); (2) anti‐CD31 antibodies (1:200, #550274, BD Biosciences; 1:200 #ab28364, Abcam; 1:200 #BBA7, R&D systems); (3) anti‐phosphorylated CD31 at tyrosine 702 (pCD31) antibody (1:200, #ab62169, Abcam); (4) anti‐ApoE antibody (1:500, #701241, Invitrogen); (5) anti‐phosphorylated tau (pTau) PHF1 antibody (1:200, from Dr. Benjamin Wolozin Lab); (6) anti‐NeuN antibody (1:1000, #ab177487, Abcam) to identify neurons; (7) anti‐GFAP antibody (1:1000, #14‐9892‐82, Fisher Scientific) for an astrocyte biomarker; (8) anti‐CD68 (1:500, #MCA1957GA, Bio‐Rad Laboratories) and anti‐Iba‐1 (1:500, #019‐19741, Wako Chemicals) antibodies for microglia biomarkers; (9) anti‐Von Willebrand Factor antibody (1:200, #ab11713, Abcam) to identify vascular damage; (10) anti‐CD3 (1:500, #ab16669, Abcam; #MAB4841, R&D systems) and anti‐CD8 (1:500, #NBP1‐49045SS, Novus Biologicals) antibodies to identify T lymphocytes; (11) anti‐CD19 antibody (1:500, #NBP2‐25196SS, Novus Biologicals) to identify B lymphocytes; and (12) anti‐CD14 antibody (1:200, #11‐0141‐82, Invitrogen) to detect monocytes; (13) anti‐Lectin antibody (1:500, #DL‐1174, Vector) and anti‐CD144 antibody (1:200, #14‐1441‐82 Invitrogen) to label vascular and endothelia.

To evaluate immunostaining results, ImageJ was used to measure total intensity after adjusting the threshold. The data obtained from two independent researchers who were blinded to the treatment groups were pooled and averaged.

### 2.6 Proximity ligation assay

A proximity ligation assay (PLA) was applied to both frozen brain sections and primary endothelial cells after fixation to investigate protein‐protein interactions (Zieba et al., 2018). The samples were washed with PBS and incubated with blocking buffer at 37°C for 1 h. To detect mCRP and CD31 interaction, samples were incubated overnight at 4°C with mouse anti‐mCRP antibody (1:50, 3H12) and either goat anti‐CD31 antibody (1:200, #AF3628, R&D Systems) for mouse samples or rabbit anti‐CD31 antibody (1:200, #ab28364, Abcam) for human samples. To detect ApoE and CD31 interaction, samples were incubated overnight at 4°C with rabbit anti‐ApoE antibody (1:200, #701241, Invitrogen) and either rat anti‐CD31 antibody (1:200, #550274, BD Biosciences) for mouse samples or mouse anti‐CD31 antibody (1:200, #BBA7, R&D Systems) for human samples. The samples were similarly incubated with mouse IgG and goat IgG or rabbit IgG and rat IgG as control groups, respectively. Proximity ligation was then conducted in situ as described by the manufacturer's instructions (#DUO92007, Sigma–Aldrich). We used the Duolink PLA probes anti‐mouse PLUS and anti‐goat MINUS to visualize mCRP/CD31 interactions and the Duolink PLA probes anti‐mouse PLUS and anti‐rabbit MINUS to visualize ApoE/CD31 interactions by Duolink in Situ Detection Reagents Orange. The samples were further incubated with Alexa Fluor 488‐ and 647‐conjugated secondary antibodies (Invitrogen) for 1 h at room temperature. Following serial washes, the samples were stained with DAPI (1:10^4^) and observed with a fluorescence microscope (ZEISS Axio Observer).

### 2.7 Microvessel isolation

Microvascular of mouse brain were isolated using dextran gradient centrifugation followed by sequential cell strainer filtrations as previously described (Lee et al., 2019). Briefly, brains were carefully removed and transfer immediately to cold MCDB131 medium. The cortex and hippocampus were dissected and all visible white matter was discarded. The isolated tissue was placed to MCDB131 medium and homogenized using a loose fit 7 ml Dounce tissue grinder. The samples were transferred into 14 ml round‐bottom centrifuge tube and then centrifuged at 2000 *g* for 5 min. The pellets were resuspended in 15% dextran/PBS (70 kDa, Sigma). The samples were thoroughly mixed and centrifuged at 10,000 *g* for 15 min, 4°C. The microvessel ‐containing pellet located at the bottom of the tubes was collected, and sequentially resuspended using 1 ml PBS. The pellet was transfer onto a 40 μm cell strainer (BD Falcon) and wash through <10 ml PBS. The microvascular remaining on top were collected in MCDB 131 medium containing 0.5% endotoxin‐, fatty acid‐ and protease free BSA and fixed by 4% PFA for further fluorescent staining analysis.

### 2.8 CD31 self‐interfering RNA (siRNA)

Primary mouse BECs were incubated with 1 μM Accell CD31‐targeting siRNA (SMART pool # E‐048240, Dharmacon) and Non‐targeting siRNA controls (# D‐001910, Dharmacon) for 72 h in Accell delivery media (# B‐005000, Dharmacon) according to the manufactural instruction. Removing medium, the BECs were treated with mCRP (10 μg/ml)/EBM‐2 basal medium for 10 h and fixed for further staining analysis. This concentration of mCRP was chosen to add into the culture based on the different concentrations and time points experiments (Figure 4b) that 10 µg/ml showed the most significant mCRP binding and CD31 phosphorylation.

### 2.9 Microvascular profile measurements

The lengths of CD31‐positive cerebrovascular profiles were measured as previously reported (Bell et al., 2012). Fixed brain sections were prepared and stained as described in the “Immunofluorescence characterization” section, followed by incubation with anti‐CD31 primary antibody (1:200, #550274, BD Biosciences; 1:200 #BBA7, R&D Systems) and Alexa Fluor 594‐conjugated secondary antibody.

In mouse brain sections, the CD31^+^ microvessels (vessels <6 μm in diameter) were selected, and their lengths were measured from the 3D structures by using the ImageJ in length analysis tool in 5 randomly selected areas in the cortex. Z‐stacks were collected at 1‐µm steps for a total imaging depth of 16 μm. The average length of CD31^+^ microvessels was expressed as μm per mm^3^ of brain tissue.

In human brain sections, we measured the length of CD31^+^ from 3D images as above and also counted the number of vessel terminals in 2D images. The lengths of blood vessels in 2D images could be affected by either vessel breakage or distortion. CD31^+^ microvessels (length >20 μm, diameter <6 μm) were selected and counted from 5 randomly selected areas for each individual sample. The average numbers were recorded per mm^2^ of brain tissue.

### 2.10 Western blots

The mouse cortex/hippocampus and postmortem frontal cortex were lysed in cold RIPA buffer supplemented with a protease and phosphatase inhibitor mixture (#78445 Thermo Fisher Scientific). For each Western blot, 40 µg of protein extract per sample was used. All samples were electrophoresed on NUPAGE 4%–12% Tris‐Glycine Gels (#XP04205BOX, Invitrogen) and transferred to PVDF membranes. The membranes were blocked with 5% milk and incubated with primary antibodies. The following primary antibodies were utilized for immunoblots: goat anti‐CD31 antibody (1:500, #AF3628, R&D Systems) for mouse, rabbit anti‐CD31 antibody (1:500, #ab28364, Abcam) for human, rabbit anti p‐CD31 (phospho Tyr 702) antibody (1:500, #ab62169, Abcam), rabbit anti p‐eNOS (Ser1177) antibody (1:500, #9571s, Cell Signaling Technology), rabbit anti‐eNOS antibody (1:500, #32027, Cell Signaling Technology), rabbit anti NF‐κB p65 antibody (1:500, #8242, Cell Signaling Technology), and rat anti‐CD144 (VE‐cadherin) antibody (1:200, #14144182, Invitrogen), mouse anti‐ β‐actin antibody (1:1000, #sc47778, Santa Cruz). After washing with Tris‐buffered saline (TBS) with Tween 20, the membranes were incubated with the appropriate HRP‐conjugated secondary antibodies in blocking buffer and imaged using a Bio‐Rad imaging station. For the quantification analysis of Western blots, a rectangular area was selected enclosing the bands of interest, and the intensity of each band was measured from the area showing the most intense signal using ImageJ. The signal intensities of all protein bands were normalized by the β‐actin band, which served as the control in this experiment.

### 2.11 Enzyme‐linked immunosorbent assay (ELISA)

Proteins were extracted from the cortex, hippocampus, or cells using RIPA buffer with a protease inhibitor mixture. Venous blood was collected by cardiac puncture and placed at room temperature for 1 h. The samples were centrifuged for 15 min at 1200*g* to obtain serum. ELISA assays were conducted for different kinds of samples. The Aβ42 (ELISA kit, **# **KMB3441, Invitrogen) and CRP (Quantikine ELISA kit, #MCRP00, R&D Systems) levels in mouse brain extracts and serum were measured by following the manufacturer's instructions. All assays were performed in duplicate, and the average of two values was used for further analysis.

### 2.12 RNA isolation and sequencing

Total RNA was isolated from dissected CD31^+^ BECs from the mouse brain using an RNA extraction kit (#74104, Qiagen). The extracted RNA was stored in RNAse‐free water at −80°C until transfer to the Boston University School of Medicine Microarray Core Facility. The RNA quality of the samples was assessed using the Agilent 2100 Bioanalyzer (Agilent Technologies). All samples passed a quality control threshold to proceed to library preparations and RNA sequencing. The libraries were prepared from total RNA enriched for mRNA using the NEBNext Poly(A) mRNA Magnetic Isolation Module and the NEBNext Ultra II Directional RNA Library Preparation Kit for Illumina (New England Biolabs) and sequenced on an Illumina NextSeq 500 instrument (Illumina). Raw reads from each library were mapped against the mouse genome build mm10 using STAR (version 2.6.0c) as described in the literature (Dobin et al., 2013). FASTQ quality was assessed using FastQC (version 0.11.7), and alignment quality was assessed using RSeQC (version 3.0.0).

### 2.13 Differential gene expression and pathway enrichment analyses

Variance‐stabilizing transformation (VST) was accomplished using the variance stabilizing transformation function in the DESeq2 R package (version 1.23.10). Differential expression was assessed using ANOVA and Wald tests as implemented in the DESeq2 R package. Correction for multiple hypothesis testing was accomplished using the Benjamini–Hochberg false discovery rate (FDR). Human homologs of mouse genes were identified using HomoloGene (version 68). All analyses were performed using the R environment for statistical computing (version 3.6.0).

Gene Set Enrichment Analysis (GSEA; version 2.2.1; Subramanian et al., 2005) was used to identify biological terms, pathways and processes that were coordinately up or downregulated within each pairwise comparison. The Entrez Gene identifiers of the human homologs of all genes in the Ensembl Gene annotation were ranked by the Wald statistic computed between mCRP and PBS within each ApoE genotype group. Ensembl human genes matching multiple mouse Entrez Gene identifiers and mouse genes with multiple human homologs (or vice versa) were excluded prior to ranking so that the ranked list represents only those human Entrez Gene IDs that match exactly one mouse Ensembl Gene. Each ranked gene list was then used to perform preranked GSEA analyses (default parameters with random seed 1234) using the Entrez Gene versions of the Hallmark, BioCarta, KEGG, Reactome, PID, transcription factor motif, microRNA motif, and Gene Ontology (GO) gene sets in the Molecular Signatures Database (MSigDB) version 7.1 (Subramanian et al., 2007). All of the gene sets were used for analysis to facilitate full reproducibility of results. We also obtained the human single‐cell RNA dataset from published study (Mathys et al., 2019) and used GSEA analyses to study gene expressions in endothelia cells in AD as described above.

### 2.14 Quantification and statistical analysis

Experimenters were blinded to the genotypes during testing. All data are presented as the mean ± standard error of the mean (SEM). Statistical analyses were performed using GraphPad Prism (version 8.0). For mouse data, datasets were analyzed for significance using Student's unpaired two‐tailed *t*‐test for two groups. Comparisons between three or more groups were conducted using one‐way or two‐way analysis of variance (ANOVA). Post hoc multiple comparisons were carried out using Tukey's test. For human data, we performed univariate analyses stratified by ApoE genotype (ApoE2‐2/2 or 2/3; ApoE3‐3/3; ApoE4‐3/4 or 4/4) or control versus AD brains. Mean ± SD was determined and ANOVA tests were conducted on variables. The correlation analysis was conducted by Pearson or Spearman test according to the distribution pattern of the data. Statistical significance was defined as *p* value < 0.05.

## 3 RESULTS

### 3.1 Peripheral mCRP causes cerebrovascular alterations in the expressions of CD31 and pCD31 in ApoE4 mouse model

Elevated serum CRP impacted the risk of AD in ApoE4 carriers in humans (Tao et al., 2018). To investigate how peripheral CRP and ApoE genotype interaction for the brain pathology, we first examined human and mouse samples with different ApoE genotypes. Both human and mice consistently showed different concentrations of serum CRP following the pattern ApoE2>ApoE3>ApoE4 (Figure S1a). We thus hypothesized that more CRP, especially the monomeric form, might stick to the brain in ApoE4 carriers. We intraperitoneally injected purified human CRP, including pCRP and mCRP, into ApoE knock‐in mice and found more CRP, particularly mCRP, deposited in mouse ApoE4 brain (Figure S1b). Consistently, i.p mCRP only caused the highest levels of mCRP in the ApoE4 brain, but produced the highest levels of mCRP in the blood of ApoE2 mice; brain mCRP and blood mCRP were negatively associated (Figure S1c,d).

**FIGURE 1:**
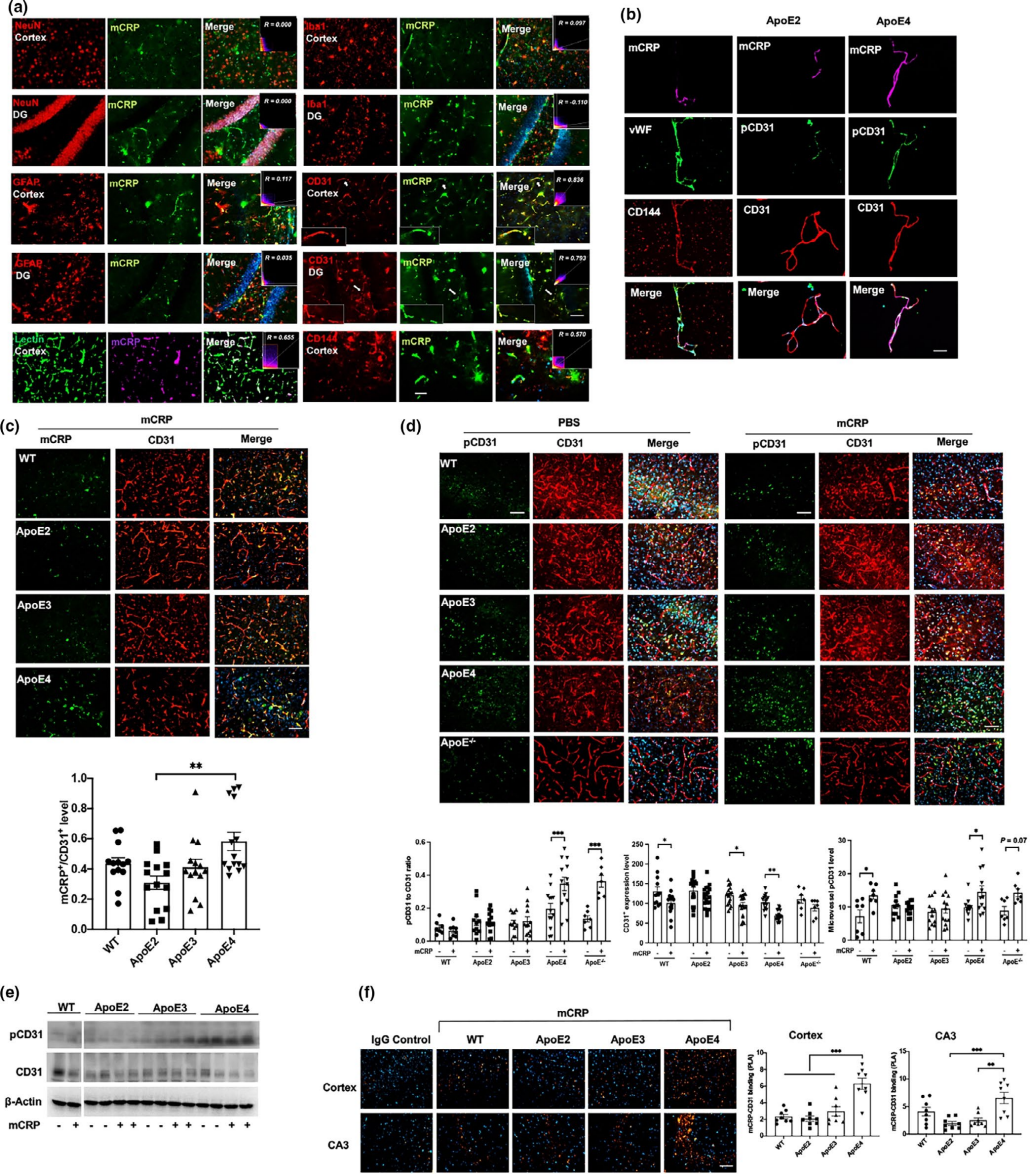
Characterization of peripheral mCRP in blood‐facing endothelial of the brain based on ApoE genotype‐dependent regulation. We used ApoE mouse models to investigate if peripheral CRP could influence cerebrovascular pathology depending on ApoE genotype. (a) We conducted intraperitoneal (i.p.). injection of mCRP into mice and characterize which cell type in the brain binds to mCRP. Double immunostaining of mCRP and different types of cell markers was conducted in the cortex and hippocampal DG region. Cell markers, including the neuronal marker NeuN, the microglial marker Iba1, the astrocyte marker GFAP, vascular marker Lectin and the endothelial cell markers CD31 and CD144, were applied individually. Correlation coefficients and *R* values were calculated for each protein pair detected and only the coefficients for mCRP and the endothelial markers including CD31, lectin or CD144 were statistically significant. (b) To further demonstrate that mCRP binds to blood‐facing endothelia in the brain, brain microvessels from ApoE2 and ApoE4 mice after treatment with mCRP were isolated and immunostained with the antibodies against mCRP (purple) and different endothelial cell components, CD144 (red), vWF (green), CD31 (red) and its phosphorylation pCD31 (green). Nuclear stained with DAPI. The bar is 20 μm. (c) We characterized the CD31‐mCRP binding across different ApoE genotypes mice after the i.p. mCRP treatment by using colocalized immunostainings. Representative images of cerebrovasculature at cortex stained with CD31 (red) and mCRP (green) and merged (yellow) are shown to observe mCRP deposits in CD31‐positive regions. The yellow fluorescence intensities were quantified and compared to show that ApoE4 mice illustrated the most intensity of CD31‐mCRP binding (*p* = 0.0016). *n* = 11–14 mice in each group. (d) Representative images of double immunostaining of phos‐CD31 (pCD31, green) and CD31 (red) and the merged images (yellow), nuclei stained with DAPI, on the cortex of WT mice, ApoE^−/−^ mice and mice expressing different ApoE genotypes after i.p. treatment with PBS versus mCRP are quantified by fluorescence intensity and shown. mCRP increased pCD31/CD31 ratio only in ApoE4 (*p* < 0.001) and ApoE^−/−^ (*p* < 0.001) mice and elevated microvessel pCD31 level in WT (*p* = 0.02), ApoE4 (*p* = 0.03), and decreased total CD31 expression in WT (*p* = 0.03), ApoE3 (*p* = 0.03) and ApoE4 (*p* = 0.001) mice. *n* = 7–19 in each group. (e) Western blots showed that mCRP increased the level of pCD31 and decreased the CD31 expression levels in the hippocampal region in ApoE4 mice but not in ApoE3 or ApoE2 mice. (f) PLA was performed on the cortex (upper panel) and hippocampal CA3 region (lower panel) to further examine the binding of mCRP and CD31 in WT mice and mice expressing different ApoE genotypes after i.p. injection of PBS versus mCRP. Positive PLA fluorescence signals are shown in orange; nuclei were stained with DAPI (blue). Quantifications of orange fluorescence were conducted. ApoE4 mice had the most mCRP‐CD31 bindings at cortex and hippocampus regions: at cortex WT versus ApoE4 *p* < 0.0001, ApoE2 versus ApoE4 *p* < 0.0001, and ApoE3 versus ApoE4 *p* = 0.0002; at CA3 ApoE2 versus ApoE4 *p* = 0.0004 and ApoE3 versus ApoE4 *p* = 0.0018. *n* = 8 in each group. Data are shown as the mean ± SEM. One or two ‐way ANOVA with Tukey's post hoc test and Pearson or Spearman correlation test were applied. *p < 0.05, **p < 0.01, ***p < 0.001, ****p < 0.0001. The scale bar is 50 μm

We further intraperitoneally injected mCRP (200 μg/kg) three times per week for 6 weeks (*n* = 15–18 in each group) to elevate peripheral level of mCRP to mimic chronic peripheral inflammation. Using antibodies against mCRP and biomarkers of different brain cell types and assessing the colocalization of fluorescence‐labeled proteins to investigate which brain cells mCRP binds to, we found that after i.p. injection, mCRP colocalized with endothelial cells marked by CD31 but not with the markers for neurons, astrocytes or microglia in the mouse brain (Figure 1a). We used two other endothelia markers to confirm that mCRP binds to endothelia and characterize different endothelial proteins for mCRP. Despite that lectin (*R* = 0.655) and CD144 (*R* = 0.570) were found to be significantly colocalized with mCRP in brain–blood vasculature, colocalized between mCRP and endothelial CD31 had the highest coefficient (*R* = 0.793; Figure 1a), suggesting two proteins could interact with each other.

We also isolated the microvessels from both ApoE2 and ApoE4 mice after the i.p mCRP treatment and used endothelial related markers, von Willebrand factor (vWF) and CD144, to confirm that more mCRP from peripheral source were colocalized with brain endothelial CD31 in ApoE4 than ApoE2 microvessels (Figure 1b). Using ApoE knock‐in brain tissues after i.p mCRP, we found more mCRP deposits in CD31‐positive endothelia regions in the ApoE4>ApoE3>ApoE2 brain (Figure 1c). Using both immunostaining (Figure 1d) and Western blot (Figure 1e and Figure S1e), we found that mCRP increased of CD31 phosphorylation (pCD31) and reduced CD31 expression in cerebrovasculature in the pattern of ApoE4>ApoE3>ApoE2 in the brain that was confirmed in the isolated brain microvessels (Figure 1b, right two columns and Figure 1d). mCRP also significantly increased pCD31 production in ApoE^−/−^ mice, which is similar as in ApoE4 mice (Figure 1d). To confirm the interaction between mCRP and endothelial CD31, the proximity ligation assay (PLA) was used to indicate that mCRP directly bound to CD31 in the cortex and hippocampus (Figure 1f) with an intensity pattern reflective of ApoE allele type (ApoE4>ApoE3>ApoE2). Taken together, the ApoE mouse model data indicate that peripheral mCRP binds to endothelial CD31 to decrease CD31 expression and increase pCD31 production in the ApoE4 brain, especially hippocampus region.

### 3.2 Peripheral mCRP causes cerebrovascular inflammation and damages in ApoE4, but not in ApoE2 or ApoE3, mice via decreasing CD31 and increasing pCD31

**FIGURE 2:**
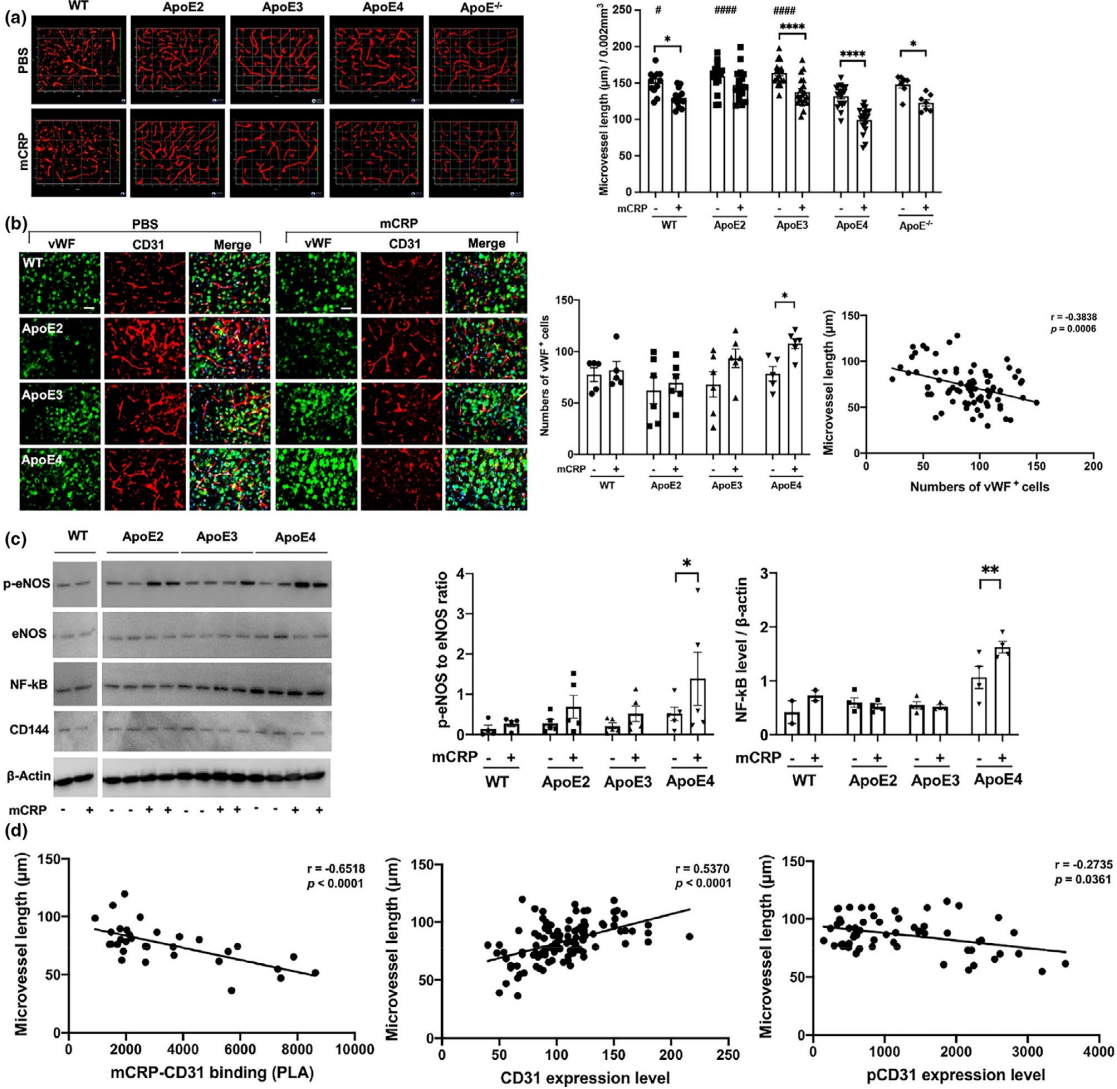
The impact of peripheral mCRP on cerebrovascular integrity in an ApoE genotype‐dependent manner. (a) After the i.p. treatment of PBS (top column) versus mCRP (bottom columns), 3D images of cortex sections stained for CD31 (red) in WT, different ApoE‐expressing and ApoE^−/−^mice were used to examine cerebrovasculature. The length of CD31‐positive microvasculature in the cortex was quantified and compared between the PBS and mCRP groups in WT (*p* = 0.01), ApoE3 (*p* < 0.0001), ApoE4 (*p* < 0.0001) and ApoE^−/−^ (*p* = 0.04) mice. # Comparison of ApoE genotypes in the PBS group, WT versus ApoE4 *p* = 0.04, versus ApoE2 *p* < 0.0001, versus ApoE3 *p* < 0.0001. *n* = 7–19 mice in each group. (b) To further examine the impact of elevated peripheral mCRP on cerebrovascular damage, we conducted immunostaining of a vascular damage biomarker, von Willebrand factor (vWF, green), and CD31 (red), in the cortex of WT vs. different ApoE knock‐in mice. Representative images of each genotype treated with PBS vehicle (left columns) and mCRP (right columns) are shown. The numbers of vWF‐positive endothelial cells in the cortex were compared among different genotype mice in the absence and presence of i.p. mCRP and showed statistical significance only in the ApoE4 group (*p* = 0.04). A negative correlation between the length of CD31^+^ microvessels and the expression of vWF in the cortex was shown (*r* = −0.38, *p* = 0.0006). *n* = 5–6 in each condition. (c) Western blots of brain tissues for different inflammatory and vascular‐related proteins, including phosphorylated eNOS (p‐eNOS), eNOS, NF‐κB and CD144, in the cortex were conducted to examine the effects of mCRP on these proteins in each ApoE genotype. Peripheral mCRP significantly increased the expression of p‐eNOS (*p* = 0.04) and NF‐κB (*p* = 0.006) only in ApoE4 mice. (d) Different correlation analyses were conducted to examine the relationship between elevated peripheral mCRP and CD31^+^ microvessels in the brain. The graphs show that the length of microvessels (y‐axis) was positively associated with the CD31 level (*r* = 0.54, *p* < 0.0001) but negatively associated with the levels of PLA (mCRP‐CD31; *r* = −0.65, *p* < 0.0001) and pCD31 (*r* = −0.27, *p* = 0.04). Data are expressed as the mean ± SEM. Two‐way ANOVA with Tukey's post hoc test and Pearson or Spearman correlation test was applied. *p < 0.05, **p < 0.01, ***p < 0.001. ^#^ *p* < 0.05, ^##^ *p* < 0.01, ^###^ *p* < 0.001. The scale bar is 50 μm

We hypothesized that mCRP may cause detrimental effects on cerebrovascular inflammation via the mCRP‐CD31 interaction and the resulting decreasing in total CD31 but increase in pCD31. We found that both ApoE^−/−^ and ApoE4 mice exhibited shorter cerebrovasculature lengths when stained with a CD31 antibody than ApoE2 and ApoE3 mice (Figure 2a top row). Elevating peripheral mCRP further shortened the length of the CD31^+^ cerebrovasculature with a severity pattern of ApoE^−/−^>ApoE4 > ApoE3, but not ApoE2, in the cortex (Figure 2a, bottom row) and hippocampus (Figure S2). This suggests that ApoE4 mice, equivalent to ApoE^−/−^, are more susceptible to cerebrovascular damage, with mCRP exacerbating these effects via decreasing CD31 expression and increasing pCD31 production.

We employed vWF (Gragnano et al., 2017), a biomarker of cerebrovascular inflammation and damage. mCRP treatment significantly increased the level of vWF only in the brains of ApoE4 mice (Figure 2b); in addition, a correlation analysis for all the experimental mice showed that the vWF level was negatively correlated with the length of CD31^+^ microvessels (*p* < 0.001; Figure 2b). mCRP treatment also increased the levels of other vascular and inflammatory factors in ApoE4 brains, such as p‐eNOS and NF‐κB, while mCRP did not influence eNOS and CD144, as shown by Western blots (Figure 2c). These data indicate that mCRP causes an elevated cerebrovascular inflammatory response more in ApoE4 brains, suggesting that ApoE2 and E3 may prevent this effect.

We conducted correlation analyses across all the ApoE genotype mice used in the experiments in the absence and presence of mCRP treatment (Figure 2d). Microvessel length in the brain was negatively associated with mCRP‐CD31 binding (*r* = −0.65, *p* < 0.0001). Consistently, microvessel length in the brain was positively associated with CD31 expression levels (*r* = 0.54, *p* < 0.0001) but negatively with pCD31 levels (*r* = −0.27, *p* = 0.04). The data again suggest that the increased mCRP‐CD31 binding in ApoE4 carriers leading to a decrease of endothelial CD31 and an increase of pCD31 production that results in greater cerebrovascular inflammation and damage.

### 3.3 mCRP promotes the extravasation of T lymphocytes into the brain of ApoE4 mice to enhance neuroinflammation and AD pathological traits

**FIGURE 3:**
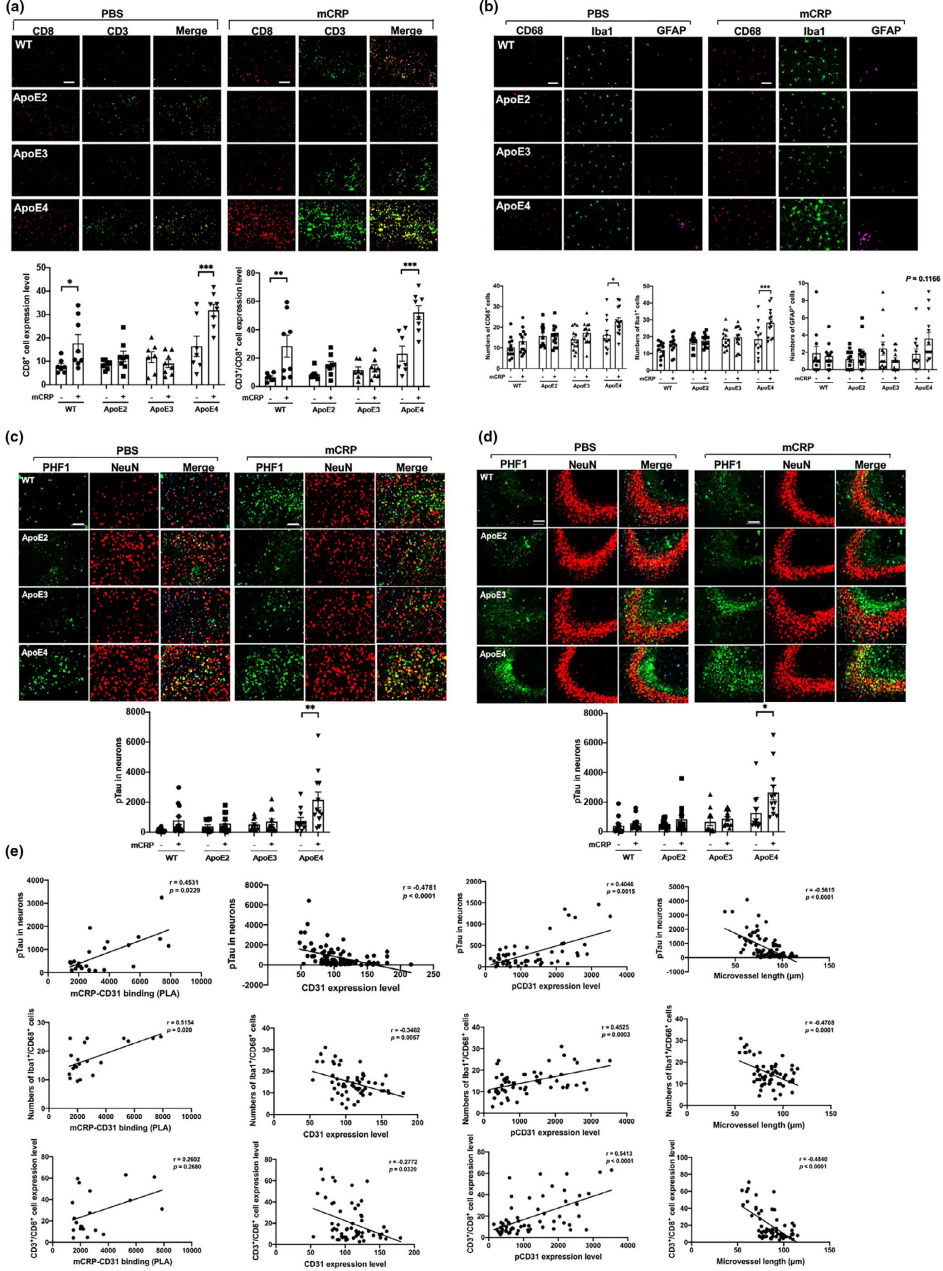
Peripheral mCRP‐induced neuroinflammation, extravasation of T lymphocytes and AD pathology traits in the ApoE4 brain. (a) We then investigated if mCRP‐induced cerebrovascular damage can migrate peripheral immune cells into the brain. Double immunostaining of CD8 (red), CD3 (green), and double‐positive cells (yellow) was performed to study the transcytosis of T lymphocytes in the cortex after i.p. treatment with PBS (left columns) versus mCRP (right columns) in WT and different ApoE knock‐in mice. Quantification of CD8^+^ T lymphocytes and CD8^+^/CD3^+^ T lymphocytes in the cortex and the comparisons between PBS versus mCRP treatment in each genotype was conducted. mCRP significantly increased the number of T lymphocytes only in the WT (*p* = 0.05) and ApoE4 (*p* = 0.001) mice. *n* = 7–8 in each condition. (b) We first investigate if elevated peripheral mCRP can induce neuroinflammation in the brain. Representative images of CD68^+^ active microglia (red), Iba1^+^ microglia (green) and GFAP^+^ astrocytes (purple) in the cortex of WT and ApoE knock‐in mice after the i.p. treatment of PBS versus mCRP are shown. Quantification of the microglial biomarkers CD68 (*p* = 0.02) and Iba1 (*p* = 0.0005) in the cortex showed significant differences between the PBS and mCRP groups only in ApoE4 mice, but the astrocyte biomarker GFAP (*p* = 0.12) only showed tendency in the ApoE4 mice. *n* = 11–14 in each group. (c and d) Representative images of i.p. mCRP‐induced neuronal tau phosphorylation (pTau) in the cortex (c) and the CA3 region of the hippocampus (d) are shown. Double immunostaining of pTau (stained with PHF1, green) and neuronal marker (stained with NeuN, red); nuclei were stained with DAPI. Quantification of PHF1 levels in NeuN‐positive cells showed statistical significance after i.p. mCRP in the cortex (*p* = 0.006) and in the CA3 region (*p* = 0.02) only in ApoE4, but not ApoE2 and ApoE3, mice. *n* =11–14 in each group. (e) Different correlation analyses using the data from all the mice used in the experiments were conducted to examine the relevance of different factors of the mCRP vs. ApoE to CD31 binding and the development of AD related pathology in the brain. The first‐row graphs show that the CD31 level (*r* = −0.48, *p* < 0.0001) was inversely related to PHF1 level in the brain; the levels of PLA (mCRP‐CD31; *r* = 0.45, *p* = 0.02) and pCD31 (*r* = 0.40, *p* = 0.002) were positively correlated with PHF1 levels; The microvessel length was negatively associated with PHF1 levels (*r* = −0.56, *p* < 0.0001). The second row graphs show that the CD31 level (*r* = −0.35, *p* = 0.007) was inversely correlated with CD68/Iba1‐positive cells in the brain, the levels of PLA (mCRP‐CD31; *r* = 0.52, *p* = 0.02) and pCD31 (*r* = 0.45, *p* = 0.003) were positively correlated with double staining of CD68/Iba1^+^ cells; The microvessel length was negatively associated with CD68/Iba1^+^ cells (*r* = −0.47, *p* = 0.0001). The third row graphs show that the number of CD8^+^/CD3^+^ T lymphocytes (*y*‐axis) was negatively associated with CD31 level (*r* = −0.28, *p* = 0.03) and positively associated with the levels of pCD31 (*r* = 0.54, *p* < 0.0001); PLA (mCRP‐CD31); CD8^+^/CD3^+^ T lymphocytes were negatively correlated with the microvessel length (*r* = −0.48, *p* < 0.0001). Data are expressed as the mean ± SEM. two‐way ANOVA with Tukey's post hoc test and Pearson or Spearman correlation test were applied. *p < 0.05, **p < 0.01, ***p < 0.001. The scale bar: 50 μm

Since damaged capillaries could migrate the immune cells, especially T lymphocytes, which are found in the AD brain (Gate et al., 2020), we examined and quantified levels of CD3^+^/CD8^+^ T lymphocytes in the cortex and hippocampus of ApoE2/3/4 mice. Figure 3a shows that elevated peripheral mCRP significantly increased the number of CD8^+^ and CD3^+^/CD8^+^ T lymphocytes in the cortex and hippocampus only in ApoE4 (*p* < 0.001) but not in ApoE3 and ApoE2 mice. Interestingly, the majority of CD3^+^ T immune cells were also pCD31‐positive cells (Figure S3a) but CD3^+^ and mCRP were not colocalized (Figure S3b), suggesting that CD31 in T lymphocytes was phosphorylated after or when CD31 mediates cell‐cell interactions to induce extravasation (Muller, 2003; Newman & Newman, 2003). In contrast, CD19^+^ B lymphocytes and CD14^+^ monocytes did not show significant changes after mCRP treatment across different ApoE genotypes (Figure S3c).

**FIGURE 4:**
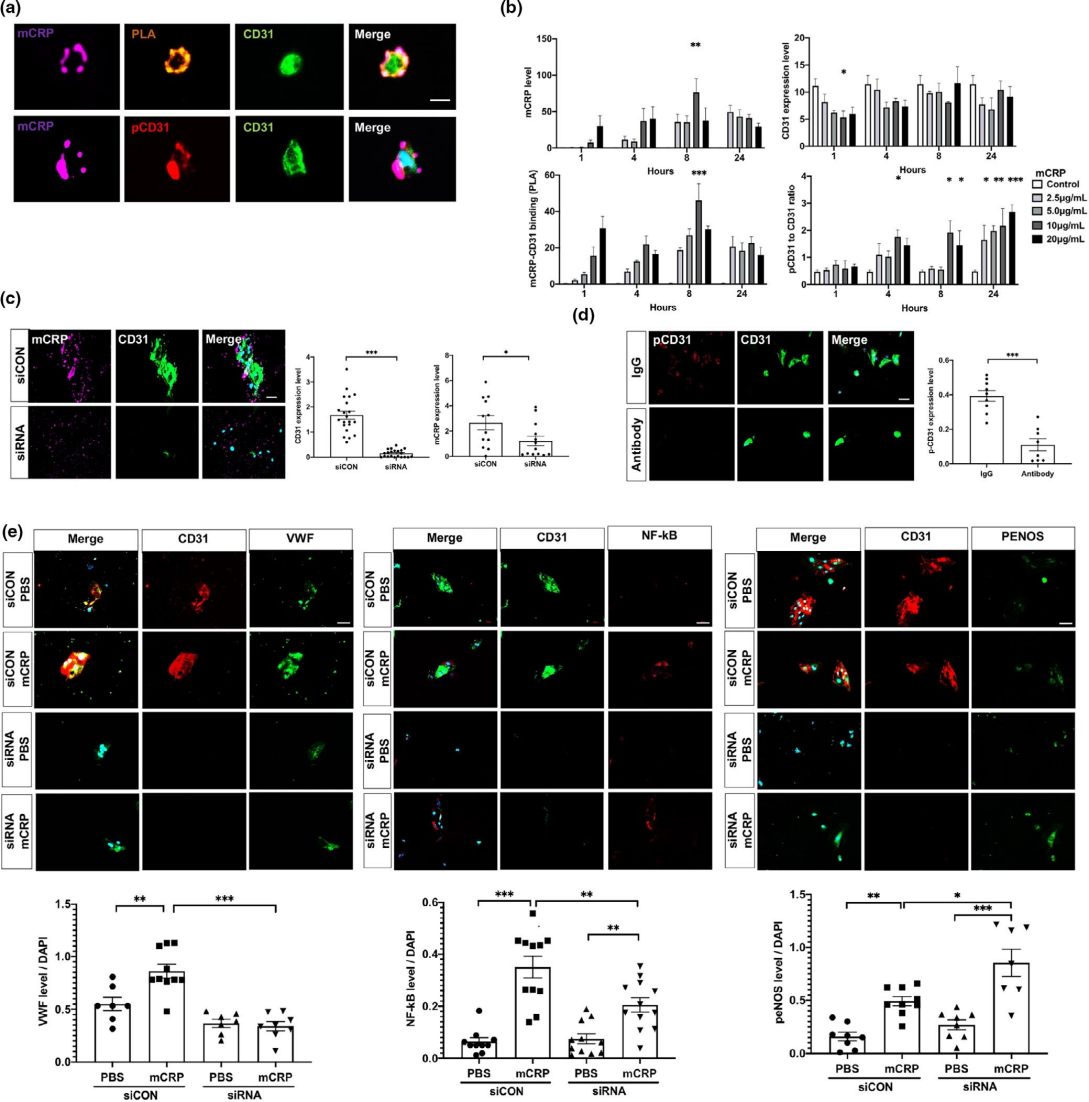
Characterization of mCRP and CD31 binding in primary brain endothelia. (a) CD31^+^ brain endothelial cells (BECs) were isolated from WT mice and cultured for 5 days. Then the in vitro BECs were incubated with mCRP (10 μg/ml) followed by detecting colocalization/binding of mCRP (purple) and CD31 (green). pCD31 (red) was detected by a specific antibody, and the nuclei were stained with DAPI. The scale bar is 10 μm. (b) To determine whether the effects of mCRP on endothelia are dose‐dependent and time‐dependent, primary CD31^+^ BECs were treated with various concentrations of mCRP for 1, 4, 8 and 24 h. Quantification is shown for the mCRP deposits on the surface of cells (upper left panel), the binding affinity of mCRP with CD31 (lower left panel), the CD31 expression level (upper right panel) and the ratio of pCD31 normalized against total CD31 (lower right panel) under different concentrations of mCRP and time course. mCRP (10 μg/ml) showed maximum binding to CD31 after 8 h of incubation (*p* = 0.0002). mCRP decreased the expression of CD31 within the first hour of incubation (*p* = 0.04) and increased the levels of pCD31 after incubation for up to 24 h (*p* < 0.001) in a dose‐dependent manner. At least three independent experiments were conducted for each condition. (c) To prove that mCRP bind with CD31 endothelial receptor to induce pCD31, primary BECs were transfected with CD31‐targeting siRNA (siRNA) to effectively knockdown CD31 expression (*p* < 0.001), compared with no‐targeting siRNA control (siCON). After adding mCRP (10 μg/ml) and incubation, fluorescence immunostainings with antibodies against mCRP (purple) and CD31 (green) were shown with the quantification. Nuclear was stained with DAPI. BECs with knockdown expression of CD31 by siRNA had lower mCRP binding ability than BECs treated with siRNA control (*p* = 0.04). (d) CD31‐specific antibody and its control Rat IgG was added to primary BECs to block CD31 before adding mCRP (10 μg/ml) to the culture. Fluorescence immunostainings with antibodies, CD31 (green) and pCD31 (red) were shown. A significant decreased pCD31 levels by mCRP were observed in BECs after preincubating with the CD31‐specific antibody to block mCRP binding, compared with IgG control (*p* < 0.001). (e) Primary BECs were transfected with CD31‐targeting siRNA (siRNA) to effectively knockdown CD31 expression (*p* < 0.001) and transfected with no‐targeting siRNA as controls (siCON) and then mCRP (10 μg/ml) was added. Representative images of primary BECs silencing of CD31 with mCRP treatment were stained with antibodies against vWF (green), NF‐κB (red), p‐eNOS (green). The nuclei were stained with DAPI. The quantifications were shown that compared with control group with intact CD31 after mCRP treatment, BECs with the siRNA knockdown CD31 failed to respond to mCRP to increase the levels of vWF (*p* < 0.001), NF‐κB (*p* = 0.003) but further increased the level of p‐eNOS (*p* = 0.005). At least three‐time experiments were conducted. Data are expressed as the mean ± SEM. Student's *t*‐test and two‐way ANOVA with Tukey's post hoc test were applied. *p < 0.05, **p < 0.01, ***p < 0.001. The scale bars are 50 and 10 μm

Additionally, elevated peripheral mCRP significantly increased the expression of Iba1 (*p* < 0.001) and CD68 (*p* < 0.05) and had a minimal effect on the expression of GFAP in the cortex of ApoE4, but not ApoE3 and ApoE2, mice (Figure 3b). Compared to the PBS treatment, the mCRP treatment significantly induced the marker of neuronal tauopathy, shown by the immunostaining of pTau with the PFH‐1 antibody, in the neurons of the cortex (*p* < 0.01; Figure 3c) and hippocampus (*p* < 0.05; Figure 3d) in ApoE4 but not in ApoE3 or ApoE2 mice. mCRP injection slightly increased the level of Aβ1‐42, another pathological marker of AD, only in the ApoE4 brain (Figure S3d).

Conducted correlation analysis using data from all the experimental mice was conducted for the brain pathology (Figure 3e). mCRP‐CD31 binding was positively correlated with the tauopathy marker (*r* = +0.45, *p* = 0.02), with the CD68^+^/Iba1^+^ microglia numbers (*r* = +0.52, *p* = 0.02), but not with CD3^+^/CD8^+^ T lymphocytes in the brain (top row). CD31 expression level was negatively correlated with the tauopathy marker (*r* = −0.48, *p* < 0.0001), with the CD68^+^/Iba1^+^ microglia numbers (*r* = −0.35, *p* = 0.007) and with CD3^+^/CD8^+^ T lymphocytes (*r* = −0.28, *p* = 0.03; middle row). In contrast to CD31, pCD31 level was positively correlated with the tauopathy marker (*r* = +0.40, *p* = 0.002), with the CD68^+^/Iba1^+^ microglia (*r* = +0.45, *p* = 0.0003) and with CD3^+^/CD8^+^ T lymphocytes (*r* = +0.54, *p* < 0.001; bottom row). As expected, microvessel length was negatively associated with the level of tauopathy marker (*r* = −0.56, *p* < 0.0001), with CD68^+^/Iba1^+^ microglia (*r* = −0.47, *p* = 0.0001) and with CD3^+^/CD8^+^ T lymphocytes (*r* = −0.48, *p* < 0.0001). Finally, CD3^+^/CD8^+^ T lymphocytes in the brain were positively associated with AD pathological markers (Figure S3e).

### 3.4 mCRP‐CD31 binding decreases CD31 expression and increases CD31 phosphorylation in the primary endothelia from brain

The above data suggest that damaged cerebrovascular damage via endothelial mCRP‐CD31 binding, decreased endothelial CD31 expression and increased its phosphorylation especially in ApoE4 carriers. To further investigate if mCRP binding to endothelial CD31 receptor, brain CD31^+^ endothelial cells (BECs) were isolated from mouse brain tissues (Yousef et al., 2018) and used to examine the binding of mCRP to CD31 and the effects of mCRP on CD31 expression and its phosphorylation. Exogenously added mCRP after incubation was colocalized with CD31, which is highly expressed on the surfaces of primary brain endothelial cells shown by colocalization and PLA (Figure 4a and Figure S4a). Figure 4b shows that after adding mCRP to the cultures, the highest level of mCRP‐CD31 binding was found at 8 h of incubation (upper left panel); CD31 expression was lowed in the first hour (upper right panel), with the highest mCRP‐CD31 binding at 8 h (lower left panel) and the production of pCD31 increasing starting at 4 h of incubation and reaching its highest level at 24 h (lower right panel) in a mCRP dose‐dependent manner (Figure S4b).

Further, after CD31 was knockdown by siRNA in primary brain endothelia, no or minimum mCRP bound to endothelia (Figure 4c), compared with no‐targeting siRNA controls. Because that siRNA almost knocked out the CD31 expression, there would be no pCD31 production (data not shown). Additionally, we also used the CD31‐specific antibody to block CD31 and found that mCRP failed to increase pCD31 production after CD31 was blocked to bind mCRP (Figure 4d). Consistently, knocked down CD31 in primary endothelia by siRNA failed to response to mCRP, resulting in little or no change of vWF and NF‐κB level but increased p‐eNOS level probably due to compensation (Figure 4e). In summary, these results further suggest that endothelial CD31 in the brain is a receptor for mCRP from peripheral inflammation.

### 3.5 mCRP is involved in the pathway regulating endothelial vascular neuroinflammation in an ApoE‐dependent manner

**FIGURE 5:**
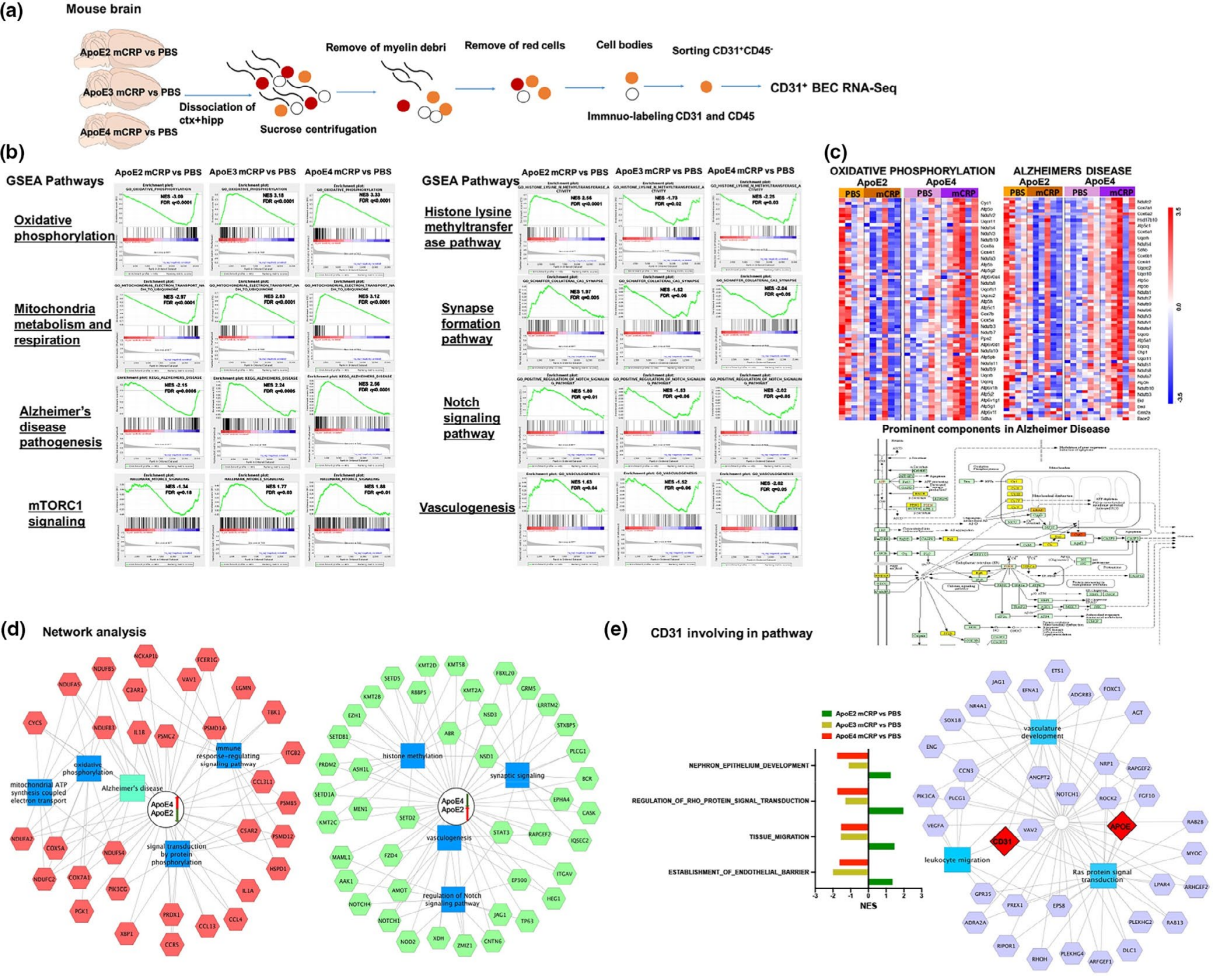
mCRP impacts the brain endothelial cell pathways in an ApoE genotype‐dependent manner. (a) The schematic showed CD31^+^ brain endothelial cells (BECs) were isolated by flow sorting and subjected to RNA sequencing from the mouse brain treated with i.p. PBS versus mCRP. (b and c) GSEA based on their differential gene expression was conducted to study the prominent pathways enriched in gene lists from different ApoE genotypes for AD pathogenesis. For the comparison between mCRP and PBS in each genotype, genes were ranked by GSEA based on their differential expression level. Four pathways, including (1) oxidative phosphorylation, (2) mitochondrial metabolism and respiration, (3) Alzheimer's disease and (4) mTORC1 signaling, were upregulated by mCRP in ApoE4 mice but were downregulated in ApoE2 mice, while ApoE3 mice were in the middle (b). In contrast, another four pathways, including (1) the histone lysine methyltransferase pathway, (2) synapse formation, (3) Notch signaling and (4) vasculogenesis, were upregulated by mCRP in ApoE2 mice but downregulated in ApoE4 mice, while ApoE3 mice were in the middle (b) The top or bottom of the ranked gene list in each ApoE comparison was evaluated using the enrichment score (green line) to determine whether a candidate pathway was significant. Black vertical lines mark positions where members of a particular pathway appear in the ranked list of genes. NES, normalized enrichment score; FDR, false discovery rate and *q* values. (c) shows the heatmaps of *z*‐scored gene intensities of the differentially expressed genes enriched in the pathways/modules of oxidative phosphorylation and Alzheimer's disease for ApoE2 and ApoE4 CD31^+^ BECs after the i.p. treatment. The key factors involved in the Alzheimer's disease pathway that overlapped with the oxidative phosphorylation pathway were ranked and labeled by different intensities of yellow color. (d) The RNAseq data were used for the functional enrichment analysis of the different signatures between ApoE4 and ApoE2 after the i.p. treatment of PBS versus mCRP by using the ToppCluster tool (FDR correction, *p* < 0.05). The red/green box represents the top genes with up/downregulated expression in the ApoE4 mCRP versus PBS comparisons and down/upregulated expression in the ApoE2 mCRP versus PBS comparisons. The genes were connected to form a hub biological process and pathway (blue box). (e) The CD31‐related pathways are listed and shown according to NES comparison between mCRP versus PBS in each ApoE genotype (left panel). The network of CD31 and ApoE together with other components involved in biological processes, including vasculogenesis, leukocyte migration and Ras signaling pathways, is illustrated (right panel)

Figure 5a schematically shows that we isolated CD31^+^ endothelial cells from mouse brain tissues (Yousef et al., 2018) and examined the effects of mCRP on CD31 receptor in the presence of different ApoE isoforms in vitro and in vivo. We used flow cytometry to isolate CD31^+^ BECs from three ApoE2, ApoE3 and ApoE4 knock‐in mice after i.p. injection of PBS or mCRP, and then used RNA sequence analysis to identify the in vivo prominent factors and important molecular pathways affected mCRP across ApoE genotypes. Here, all the genes were ranked by the Wald‐test statistic computed between mCRP and PBS for each ApoE genotype, followed by GSEA analysis (Figure 5b). We identified sets of genes that are coordinately up and downregulated with respect to expression changes between mCRP and PBS in each ApoE genotype group. The most highly enriched gene sets upregulated in the cerebral endothelia of ApoE4 mice by elevated mCRP reflected changes in pathways including (1) oxidative phosphorylation, (2) mitochondria metabolism/respiration, (3) mTORC1 signaling, and (4) Alzheimer's disease; in contrast, these pathways were downregulated by mCRP in the endothelia of ApoE2 brains. On the other hand, mCRP significantly upregulated other pathways in the endothelia of ApoE2 brain, including (1) epigenetic histone methylation, (2) synapse formation, (3) Notch signaling and (4) vasculogenesis; in contrast, mCRP downregulated these pathways in the endothelia of ApoE4 brains. The enrichment scores of these pathways in the ApoE2 group were inversely correlated with those in ApoE4, while the ApoE3 comparisons yielded intermediate enrichment scores (Figure S5a).

Figure 5c shows the heatmaps of the top differentially expressed genes in the pathways of oxidative phosphorylation and Alzheimer's disease in the CD31^+^ BEC of ApoE2 and ApoE4 mice treated with PBS versus mCRP. Most of these genes, which are involved in the oxidative phosphorylation pathway and also play a key role in AD pathogenesis, showed increased expression in ApoE4 mice treated with mCRP but reduced expression in ApoE2 mice treated with mCRP. We also performed functional network analysis according to the ranked gene list (Figure 5d and Figure S5b). ApoE4‐specific signature genes are featured prominently in the processes of oxidative phosphorylation (CYCS, COX5A/7A), mitochondrial ATP synthesis (NDUFA and NDUFB, NDUFC), the immune response (IL1A, IL1B, CCL3L1) and protein phosphorylation (PRDX1, PIK3CG). ApoE2‐specific signature genes functioned mainly in histone methylation (NSD1, SETD2), synaptic signaling (KMT2A, STAT3), Notch signaling (NOTCH1, EP300) and vasculogenesis (ZMIZ1, XDH). We extracted CD31‐associated gene ontologies and found that CD31‐associated signaling pathways were upregulated in ApoE2 but downregulated in ApoE4 and ApoE3 (Figure 5e). As there is some evidence that support Src family kinase to mediate CD31 tyrosine phosphorylation (Newman & Newman, 2003), we found that Src kinase Fgr mRNA expressive level was elevated in ApoE4‐mCRP group (Figure S5c). Lastly, the network analysis indicated that both CD31 and ApoE together were involved in the biological processes of vascular development, leukocyte migration and Ras signal transduction (Figure 5e), suggesting that both CD31 and ApoE are linked in vascular‐related pathways that are susceptible for AD pathogenesis.

### 3.6 Investigating the influences of ApoE genotype on endothelial mCRP‐CD31 binding

**FIGURE 6:**
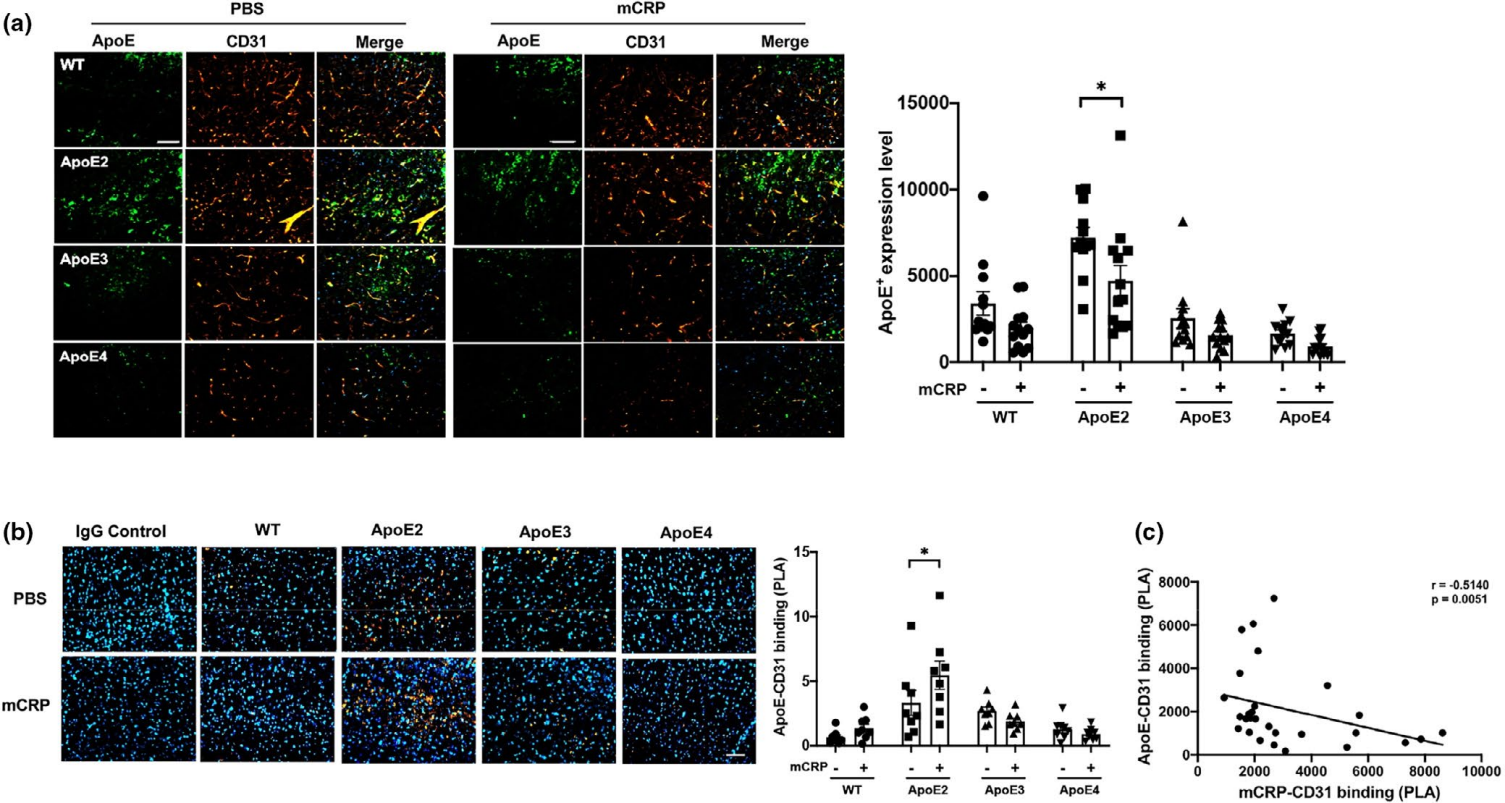
The binding of ApoE protein to CD31^+^ endothelial cells across ApoE genotype mice. (a) Because the endothelial CD31‐mCRP bindings were different depending on ApoE genotypes, we hypothesized the association between ApoE protein and CD31. Representative images of double immunostaining of ApoE (green) and CD31 (red) and the merged images (yellow) of the cortex sections of WT vs. different ApoE knock‐in mice are shown. The ApoE^+^ fluorescence intensity was compared between PBS and mCRP‐treated mice and reached a significant difference only in the ApoE2 mice (*p* = 0.02). *n* = 11–14 mice in each group. (b) Further, PLA was applied to detect the binding of ApoE and CD31 on cortex sections from different ApoE genotype mice treated with PBS versus mCRP. Positive PLA fluorescence signals are shown in orange, and nuclei are stained with DAPI (blue). Quantifications of orange fluorescence intensity of ApoE‐CD31 binding in the cortex are shown and again reached a significant difference only in ApoE2 mice by mCRP treatment (*p* = 0.04). (c) We found that PLA (mCRP‐CD31 binding) levels were negatively correlated with PLA (ApoE‐CD31 binding) in the brain (*r* = −0.51, *p* = 0.005). n = 8 in each group. Data are expressed as the mean ± SEM. Student's *t*‐test and two‐way ANOVA with Tukey's post hoc test and Pearson or Spearman correlation test were applied. *p < 0.05, **p < 0.01, ***p < 0.001. The scale bars are 50, 20, and 10 μm

To address, if CD31 is linked with ApoE and why mCRP‐CD31 binding and responses are ApoE genotype dependent, we examined brain levels of ApoE protein across ApoE genotypes and found that ApoE2 mice had the highest level of ApoE protein regardless the mCRP treatment (Figure 6a). Since ApoE2 mice had the highest level of ApoE protein in the brain, while ApoE4 mice had the lowest level (Figure 6a), we hypothesized the possible binding of ApoE protein to CD31. We used PLA and did find that peripheral i.p. injection of mCRP significantly increased ApoE‐CD31 binding only in the brains of ApoE2, but not in ApoE3 and ApoE4, mice (Figure 6b), an opposite direction from the mCRP‐CD31 binding (Figure 1). We found that mCRP‐CD31 colocalization/binding was negatively correlated with ApoE‐CD31 colocalization/binding (*r* = −0.51, *p* = 0.005) in the brain (Figure 6c). No binding/colocalization between ApoE and mCRP was observed (Figure S6). Thus, it is possible that elevated peripheral mCRP and ApoE protein competitively bound to CD31 to regulate CD31 expression and CD31 phosphorylation for AD pathogenesis in the brain.

### 3.7 Cerebrovascular alterations in the levels of mCRP, CD31 and phosphorylated CD31 in human AD brains

**FIGURE 7:**
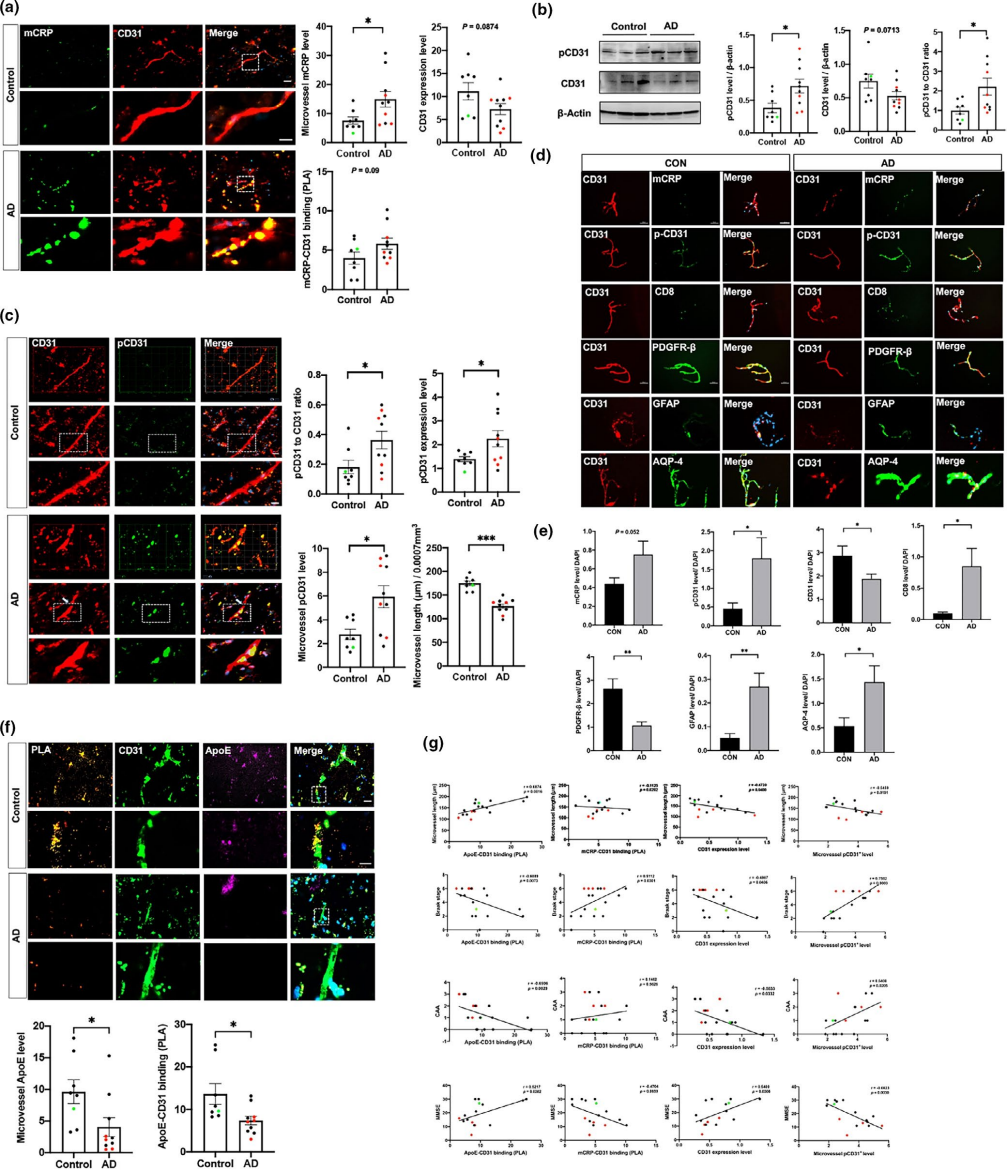
Characterization of mCRP in blood‐facing endothelial of cerebrovasculature of AD brains. The human temporal cortex of healthy controls (*n* = 8) and AD patients (*n* = 10) were used for immunostaining. (a) Representative images of CD31^+^ capillaries (micorvessels; red), mCRP (green) and the merged images (yellow) in the temporal cortex are shown and quantified. AD brains had higher mCRP signal in microvessels (yellow; *p *= 0.01) and tended to have lower levels of microvessel CD31 expression (red; *p* = 0.09) than control brains. AD brains tended to have higher levels of binding between mCRP and CD31 detected by using PLA (*p* = 0.09) than control brains. (b) Western blots for pCD31 and CD31 expression in the temporal cortex were performed and quantified after normalization against β‐actin and compared between AD and controls. AD brains had higher levels of pCD31 (*p* = 0.02), tended to have lower levels of total CD31 (*p* = 0.07), and had a higher pCD31/CD31 ratio (*p* = 0.03) than control brains. (c) Double immunostaining of temporal cortex sections with CD31 (red), pCD31 (green) and the merged images (yellow) were conducted to examine CD31 phosphorylation and microvessel integrity. AD brains had higher brain pCD31 levels (*p* = 0.05), pCD31/CD31 ratio (*p* = 0.03), and microvessel pCD31 (*p* = 0.01). Measurement of the lengths of CD31^+^ microvessels from 3D images revealed shorter lengths in AD brains than in controls (*p* < 0.001). (d) To further confirm that low expression of CD31 and high pCD31 in AD cerebrovasculature, we isolated microvessels from human cortex. Immunofluorescence analysis of isolated brain microvessels from controls and AD cases were stained with antibodies for molecular components CD31 (red color), merged with green color of different antibodies against other proteins. The bar is 50 μm. (e) The quantitation and comparisons for these proteins were conducted. AD capillaries had higher levels of mCRP (*p* = 0.05), pCD31 (*p* = 0.04), CD8 (*p* = 0.04), GFAP (*p* = 0.006) and AQP‐4 (*p* = 0.02), but lower levels of CD31 (*p* = 0.03) and PDGFR (*p* = 0.005), than control capillaries. (f) Human study was conducted. PLA was also performed to detect interaction/binding between ApoE and CD31 in the temporal cortex of control and AD brains. Representative images of positive PLA (orange), ApoE (green) and CD31 (purple) staining are shown. The levels of microvessel ApoE and ApoE‐CD31 were quantified and compared. In opposite from mCRP level and mCRP‐CD31 binding, AD brains had significantly lower levels of microvessel ApoE expression (*p* = 0.02) and ApoE‐CD31 binding (*p* = 0.03) than control brains. (g) Correlation analyses were conducted to investigate the mCRP‐ApoE‐CD31 pathway and brain AD pathology in humans. In the first row, the microvessel length (*y*‐axis) was negatively associated with pCD31 (*r* = −0.55, *p* = 0.02), CD31expression (*r* = −0.47, *p* = 0.048) and positively associated with ApoE‐CD31 (*r* = 0.69, *p* = 0.002), but not associated with mCRP‐CD31 binding. In the second row, the Braak stage (*y*‐axis) was negatively associated with CD31 expression (*r* = −0.49, *p* = 0.04), ApoE‐CD31 binding (*r* = −0.61, *p* = 0.007) and microvessel length (*r* = −0.70, *p* = 0.001); in contrast, the Braak stage was positively associated with mCRP‐CD31 binding (*r* = 0.51, *p* = 0.03) and microvessel pCD31 expression (*r* = 0.76, *p* = 0.0003). In the third row, the CAA (*y*‐axis) was negatively associated with CD31 expression (*r* = −0.50, *p* = 0.03), ApoE‐CD31 binding (*r* = −0.66, *p* = 0.003); in contrast, CAA was not associated with the level of mCRP‐CD31 binding but was positively associated with microvessel pCD31 expression (*r* = 0.54, *p* = 0.02). In the fourth row, the Mini‐Mental State Exam (MMSE) score (*y*‐axis) was positively associated with CD31 expression (*r* = 0.54, *p* = 0.03), the level of ApoE‐CD31 binding (*r* = 0.52, *p* = 0.04); in contrast, MMSE score tended to be negatively associated with mCRP‐CD31 binding (*r* = −0.47, *p* = 0.07) and significantly associated with the level of microvessel pCD31^+^ cells (*r* = −0.69, *p* = 0.003). Data are expressed as the mean ± SEM. Student's *t*‐test and Pearson or Spearman correlation tests were used. *p < 0.05, **p < 0.01, ***p < 0.001. The scale bars are 100, 20, and 10 μm

**FIGURE 8:**
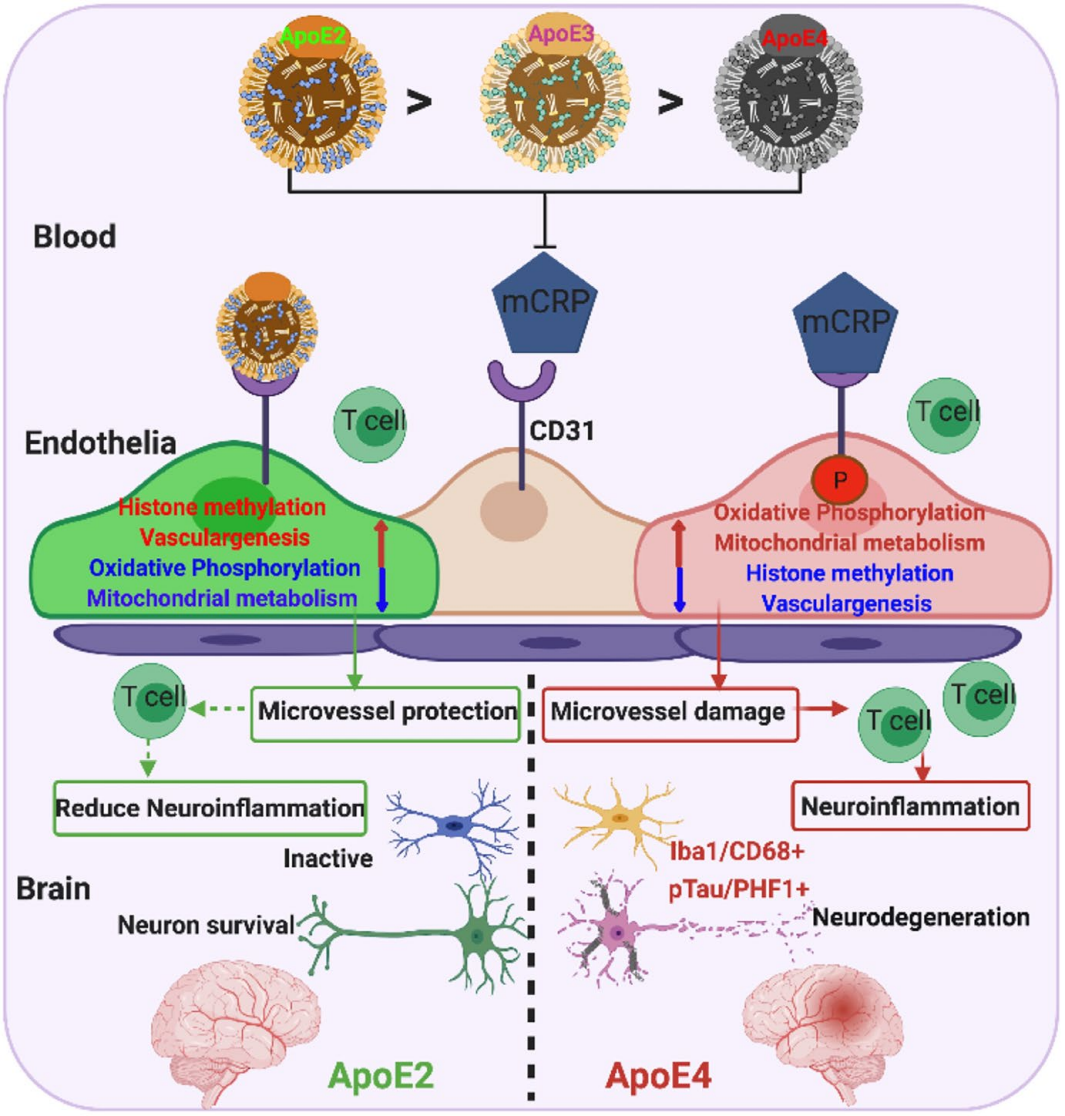
A model illustrating the differential responses of ApoE2 vs. ApoE4 carriers to mCRP and the differential regulation of mCRP‐induced cerebrovascular neuroinflammation leading to AD pathogenesis in the brain. This study demonstrated a novel pathological mechanism, the competition of ApoE and mCRP to CD31binding, for cerebrovascular neuroinflammation resulting in an early stage of AD pathogenesis in the brain. During the chronic stage of peripheral inflammation, pCRP proteins disassociate into mCRP. mCRP binds to CD31 on blood‐facing endothelia to increase CD31 phosphorylation (pCD31), cause damage to the cerebrovasculature and induce extravasation of T lymphocytes into the brain, leading to AD pathogenesis (ApoE4>ApoE3>ApoE2). This process is antagonized by ApoE‐CD31 binding (ApoE2>ApoE3>ApoE4) to block mCRP‐CD31 binding and differentially regulate pathways (mitochondrial function, epigenetics and vasculogenesis) to intervene in the neurodegenerative process of AD

To investigate if mCRP‐CD31 binding vs. ApoE‐CD31 binding are relevant to AD pathology, we used human temporal lobe tissues from 8 healthy controls and 10 AD patients (Figure 1, 2, 4, 5, 7, 8). We observed stronger mCRP immunostaining in the CD31^+^ cerebrovasculature of AD temporal cortex sections than in those of healthy controls (Figure 7a). The PLA was further applied to investigate that more mCRP directly bound to surface CD31 in AD sections than in control sections (Figure 7a and Figure S7a,b), suggesting that mCRP through endothelial CD31 for AD pathology.

Next, Western blots of brain tissues showed that total CD31 levels tended to be lower, but there was a higher average level of phosphorylated CD31 (pCD31), in AD cortex tissue than in control cortex tissue (Figure 7b). Consistently, the images of immunolabeled brain sections show that pCD31 labeling in cerebrovasculature was significantly higher in AD brains than in control brains (Figure 7c). In addition, AD brains exhibited CD31^+^ cerebrovascular with shorter lengths (Figure 7c) and more vessel termini (Figure S7c) than controls. We isolated microvessels, which are primarily comprised of endothelial cells marked by CD31, also including pericytes, pre‐capillary arteriolar smooth muscle cells and astrocyte foot processes, from human cortex (Figure 7d). It is shown that AD microvessels had higher levels of mCRP (*p* = 0.05), pCD31 (*p* = 0.04), CD8 (*p* = 0.04), GFAP (*p* = 0.006) and AQP‐4 (*p* = 0.02), but lower levels of CD31 (*p* = 0.03) and PDGFR (*p* = 0.005), than control capillaries (Figure 7e).

We further studied human brain tissues for the possible binding between ApoE and CD31 and found that the ApoE level in microvessels was significantly lower in human AD brains compared with the one in control brains; using double staining and PLA assays, we found that AD brains had lower levels of ApoE‐CD31 binding than the control brains (Figure 7f). Of note, ApoE4 carriers have lower ApoE in microvessel and the binding with CD31 (red dots). To verify the relationship between the ApoE‐mCRP‐CD31 pathway in cerebrovasculature and the neuropathological and clinical phenotypes of AD in humans, we performed correlation analyses (Figure 7g). ApoE‐CD31 binding, in contrast, mCRP‐CD31 binding, were opposite associated with the microvessel length, with Braak stage of AD pathology, with CAA and with MMSE scores (left two columns). Additionally, CD31 expression and pCD31 level were opposite associated with the microvessel length, with Braak stage of AD pathology, with CAA and with MMSE scores (right two columns). Taken together, peripheral mCRP has an inflammation actions on cerebrovascular by disrupting the endothelial pathways in the presence of ApoE4 to cause extravasation of immune cells into the brain leading to AD pathogenesis.

## 4 DISCUSSION

This study demonstrates a novel pathological pathway, the ApoE‐mCRP‐CD31 pathway, in the blood‐facing endothelia that differentially affects cerebrovasculature as an AD risk in an ApoE allele‐dependent fashion, and that is likely exacerbated when peripheral chronic inflammation occurs (Figure 8 schematic). We find that mCRP binds to endothelial CD31 receptor, probably competing with ApoE protein for CD31 which had ApoE4 equivalent to ApoE knockout with least protection (Figures 1, 2 and 6). Elevated peripheral mCRP during chronic inflammation leads to decreased CD31 expression and increased phosphorylation of CD31 (pCD31), caused cerebrovasculature damage marked by shortened capillary lengths (Figure 2), leading to the extravasation of T lymphocytes and the development of some AD pathological markers particularly in the ApoE4 brain (Figure 3).

Peripherally elevated mCRP caused the highest mCRP deposits in the ApoE4 brains compared to ApoE3 and ApoE2 brains, probably leading to a lower blood CRP level in ApoE4 carriers (Figure S1). While these ApoE mouse models do not have typical pathologies for either tau or Aβ, our study illustrated that mCRP plays a role in causing cerebrovascular inflammation, which is an early‐stage risk for AD, through in an ApoE4‐related pathway (Figures 2, 3 and 5). In parallel, human AD brains have a high level of mCRP‐CD31 binding in the cerebrovasculature (Figure 7). Consistently, our recent study shows that higher plasma CRP was more significantly associated with total Tau and p‐Tau level in ApoE4/4 carriers than Aβ (Tao et al., 2021). Although other studies have shown that mCRP colocalizes with amyloid pathology in the human AD brain (Slevin et al., 2015, 2017), amyloid pathology and tau pathology in AD could be regarded as independent as well as feedback each other pathological events (Ittner et al., 2010; Kametani & Hasegawa, 2018). ApoE4 is shown to have enhancing effects to induce neuroinflammation (Li et al., 2015), chronic peripheral inflammation induces cytotoxic effects and increased the occurrences of neuroinflammation and the severity of neurodegeneration (Kempuraj et al., 2017). Additionally, CRP levels are known to increase with age (Tao et al., 2018), and mCRP has been shown to play a role in the pathogenesis of peripheral vascular diseases including cardiovascular diseases (Wang et al., 2015) and poststroke inflammation (Slevin et al., 2010).

Our study demonstrates and argues that endothelial CD31 is a receptor for mCRP based on the following findings: (1) high colocations and PLA result of mCRP and CD31 (Figure 1), (2) mCRP‐CD31 bound and increased pCD31 production in a dose‐ and time‐dependent manner (Figure 4b) and (3) knocking down CD31 by siRNA eliminated mCRP binding and activities (Figure 4c–e). mCRP‐CD31 binding and pCD31 were linked with shortened CD31^+^ cerebrovasculature predominantly in the ApoE4 brain (Figures 1 and 2) and were linked with CAA in humans (Figure 7), the major cerebrovascular pathology in human AD (Greenberg et al., 1995). As mCRP‐induced neuroinflammation (Figure 3) in ApoE4 mice, phosphorylation of the CD31 cytoplasmic domain (Jackson et al., 1997) has been shown to limit the inhibitory function of CD31 to inflammatory reactions (Douaisi et al., 2017; Privratsky & Newman, 2014). Consistently, elevated mCRP caused the changes of several cerebrovascular biomarkers. As a factor released from damaged vasculature (Gragnano et al., 2017), the levels of vWF were negatively correlated with vascular length and significantly increased by mCRP selectively in the ApoE4 brain (Figure 2b) and NF‐κB and vWF are the downstream deteriorating factors, in the ApoE‐mCRP‐CD31 pathway for cerebrovascular inflammation (Figures 2c and 4e). Increase vWF expression was associated with the blood vessel abnormalities in the neurodegeneration mouse model and AD patients, which suggested the endothelial cells contribute to angiogenesis and immune repose in AD pathogenesis (Lau et al., 2020). As a proinflammatory transcription factor, NF‐κB has an essential role in inflammation in endothelial cells and the activation of NF‐κB was responsible to increase eNOS mRNA expression to response to shear stimulation (Grumbach et al., 2005). eNOS, especially p‐eNOS, is a protective factor in the vasculature and dementia by being responsible for most of the vascular nitric oxide (NO) produced that is a feedback to inhibit NF‐κB actions (Förstermann & Münzel, 2006; Mount et al., 2007). Paradoxically, mCRP also induced the p‐eNOS level particularly in ApoE4‐expressing cells and further increased the p‐eNOS level in CD31 knockdown endothelia (Figure 4e). One possibility is that mCRP could bind a receptor other than CD31 to increase p‐eNOS production. In addition, another study shows that ApoE4‐positive endothelial cells derived from human stem cells have higher levels of Aβ, vWF and cytokines than ApoE3 endothelial cells, suggesting that the ApoE isoforms affect endothelial cell function (Rieker et al., 2019). CD31^+^ endothelial cells are also shown to be vascular stem cells (Jankowska‐Steifer et al., 2015) and probably are the precursors of pericytes for BBB (Yamamoto et al., 2017).

Our study discovered that peripheral mCRP increased the number of T lymphocytes and possibly monocytes in the ApoE4 brains (Figure 3a), probably through enhanced migrating of peripheral immune cells into the brain. This finding is supported by a recent paper demonstrating that the number of clonally expanded CD8 T cells is high in the cerebrospinal fluid of AD patients (Gate et al., 2020). Although CD31 is expressed in both endothelial and immune cells in peripheral tissues (Cheung et al., 2020; O'Brien et al., 2003; Muller, 2003; Newman & Newman, 2003), mCRP was found to bind endothelial CD31 in ApoE4 brains (Figure 1) but not to CD31 in migrated T lymphocytes in the brain (Figure S3b). Since CD31 expressed in peripheral endothelial cells plays roles in regulating endothelial peripheral vascular junction/integrity (Douaisi et al., 2017; Privratsky & Newman, 2014), our study suggests that mCRP modulates extravasation of T lymphocytes mainly through binding to brain endothelial CD31 based on the following results: (1) mCRP increased CD31 phosphorylation and decreased the expression of CD31 in primary endothelia (Figure 4). Lack of CD31 is associated with increased mononuclear leukocyte translocation into the CNS in a mouse model of experimental autoimmune encephalomyelitis (EAE; Graesser et al., 2002). (2) Endothelial CD31 protein expression is probably reduced in the AD brain (Figure 7). Endothelial culture from AD patients increase monocyte transmigration compared to those from normal controls, and Aβ treatment enhances this transmigration (Giri et al., 2002), as microvascular endothelia contraction can be accompanied by leukocyte extravasation in peripheral tissues (Reed, 2003). mCRP enhanced the level of pCD31 in both endothelia cell by direct interaction (Figure 4), and the immune cells (especially T cells) without directing binding in the ApoE4 brain (Figure S3a,b). Endothelial CD31 was activated by homophilic interactions between leukocyte CD31 and vascular endothelia CD31 to induce T‐ immune cell extravasation into the brain under inflammatory conditions (Muller et al., 1993). We assume CD31 in T lymphocytes was phosphorylated after or when mCRP stimulated endothelial CD31 induce the inflammatory to active trans‐endothelial migration od leukocytes, especially into ApoE4 brain (Figure S5b). pCD31 has a critical role to regulate the transmigration of monocytes through the endothelial BBB (Giri et al., 2002) and phosphorylation of the CD31 cytoplasmic domain (Jackson et al., 1997) in immune cells has been shown to limit the inhibitory response of CD31 to inflammatory reactions.

It is intriguing that the molecular pathways triggered by mCRP‐CD31 binding demonstrated oppositional regulation in ApoE4 vs. ApoE2, but the similar alternations between ApoE4 and ApoE3 endothelia (Figure 5). Although blood‐facing endothelia in the brain express little ApoE, endothelia can be exposed to, and influenced more by, ApoE protein in blood, and human studies show that lower plasma concentrations of ApoE are associated with brain AD pathology (Lazaris et al., 2015). Notably, after mCRP stimulation *in vivo*, ApoE4 BECs showed significantly increased, but ApoE2 BECS decreased, gene expression related to four pathways linked to neurodegenerative processes (1) oxidative phosphorylation, (2) mitochondrial metabolism, (3) mTORC1 signaling and (4) AD pathology (Figure 5b,d,e). On the other hand, ApoE2 BECs showed upregulation, but ApoE4 BECs showed downregulation, of four other pathways more commonly associated with neuroprotection (1) histone methylation‐epigenetics, (2) synapse formation, (3) Notch signaling and (4) vasculogenesis (Figure 5b,d). Compared with risk ApoE4 and protective ApoE2 for AD, mCRP exposure in ApoE3 BECs showed similar mRNA profiles to the ones of ApoE4 and cannot explain that ApoE3 is a lower risk carrier for AD compared to ApoE4 under chronic inflammation. However, mRNA is only one aspect of cellular processing responding to mCRP pursued in this research; the future study should address if the relevant protein levels distinguish ApoE3 and ApoE4. Interestingly, Rho signaling was linked with both CD31 and ApoE in endothelial network modules of vascular development and leukocyte migration by mCRP treatment (Figure 5e). Deficient regulation of Rho signaling contributes to impaired endothelial barrier integrity and elevated p‐eNOS levels and that Ras/ERK signaling plays an important role in T cell migration, which is regulated by eNOS (Cheung et al., 2020). RhoA/Rho kinase (ROCK) is also involved in eNOS function (Ibiza et al., 2008), as its activation decreases eNOS expression to disrupt endothelial barriers (Laufs & Liao, 1998). Signals mediated by CD31 in the endothelium are both necessary and sufficient to prevent endothelial cell death and confer immune privilege to protect the vascular endothelium after inflammatory attacks (Cheung et al., 2015). These data are consistent with several published results on AD pathological pathways. Oxidative phosphorylation provides an energy‐generating pathway relying on mitochondria for ATP production to maintain cerebral vasculature (Busija & Katakam, 2014). Mitochondrial dysfunction in endothelial cells has been implicated in mediating BBB failure in degenerative disorders, such as AD (Onyango et al., 2016). A study indicated that ApoE deficiency plus adverse factors could alter histone lysine methylation modifications in vascular endothelial cells (Alkemade et al., 2010). It has been shown that Notch pathway genes, such as Notch1 and Jag1, are downregulated during the response to vascular injury (Gridley, 2007). While mCRP activates Notch to promote angiogenesis through PI3K (Boras et al., 2014), in contrast, after Notch signaling deactivation, endothelial cells lose their barrier function, which leads to endothelial hyperpermeability, leakage and inflammatory responses (Tian et al., 2017). These results suggested that mCRP binds with the extracellular domain of CD31, via phosphorylated modification probably by Src kinase Fgr to activate mitochondrial and inflammation pathways and suppress vasculogenesis (Newman & Newman, 2003). Future research is needed to investigate the direct ligand mCRP with its receptor CD31 binding by using FERT and chemical cross‐linking.

In summary, and as illustrated in Figure 8, our study provides some evidence for an ApoE‐mCRP‐CD31 pathway in blood‐facing endothelia in brain that can regulate responses to mCRP in peripheral chronic low‐grade inflammation and impact on cerebrovascular inflammation. Probably through different abilities to compete with mCRP for CD31, ApoE4 exhibits effects on the cerebrovasculature that are more detrimental than ApoE3 and ApoE2. In the ApoE4 background equivalent to ApoE knockout, mCRP increases CD31 phosphorylation to promote cerebrovascular damage via disrupting mitochondrial metabolism, enhancing oxidative phosphorylation and increasing the immune response, causing cerebrovascular neuroinflammation leading to the formation of AD pathological markers. In contrast, ApoE3/2 may protect the brain via a mechanism that involves upregulating epigenetic modifications, Notch signaling and vasculogenesis under inflammation condition. Given the high frequency at which elderly people experience peripheral inflammatory attacks and develop chronic low‐grade inflammation, which results in the formation and release of mCRP, these data may provide one perspective on why some, but not all, ApoE4 carriers develop AD by the age of 90, and why ApoE2 is protective against the disease (Tanzi, 1999; Tao et al., 2018).

## CONFLICT OF INTEREST

The authors declare no biomedical financial interests or potential conflicts of interest.

## AUTHOR CONTRIBUTIONS

Z.Z. and W.Q. designed the research; Z.Z., H.N. and Q.G. performed the research; Z.Y., J.Y., S.Z., H.T. and Q.L. contributed to data analysis; I.R., B.W. and L.P. provided the reagents; Y.A. contributed to RNA sequencing; J.B., B.W. and A. E. contributed to project suggestion; T. S. provide the human samples. J.H., Q.T. and X.Z. analyzed the human data; Z.Z. and W.Q. were responsible for results discussion as well as manuscript preparation.

## Supporting information

Fig S1‐S7

Fig S5E

Table S1

## Data Availability

The data that support the findings of this study are available from the corresponding author upon reasonable request.
