# Supplementary material for: Monomeric C‐reactive protein via endothelial CD31 for neurovascular inflammation in an ApoE genotype‐dependent pattern: A risk factor for Alzheimer’s disease?
Source: Aging Cell. 2021 Oct 23;20(11):e13501. doi: 10.1111/acel.13501 (PMC8590103; doi:10.1111/acel.13501)

**Supplementary Materials**

Monomeric C-reactive protein via endothelial CD31 for neurovascular inflammation in an ApoE genotype dependent pattern: a risk factor for Alzheimer’s disease?

Zhengrong Zhang^1^, Hana Na^1^, Qini Gan^1^, Qiushan Tao^1^, Yuriy Alekseyev^2^, Junming Hu^3^, Zili Yan^1^, Jack B. Yang^1^, Hua Tian^1,10^, Shenyu Zhu^1^, Qiang li^1,11^, Ibraheem M. Rajab^12^, Jan Krizysztof Blusztajn^4^, Benjamin Wolozin^1^, Andrew Emili^5^, Xiaoling Zhang^3^, Thor Stein^4,6,8^, Lawrence A. Potempa^12^ and Wei Qiao Qiu^1,6,7*^

^1^ Department of Pharmacology and Experimental Therapeutics, ^2^ Microarray and Sequencing Core Facility, ^3^ Department of Medicine, ^4^ Department of Pathology and Laboratory Medicine, ^5^ Department of Biochemistry, ^6^ Alzheimer’s Disease Center, ^7^ Department of Psychiatry, Boston University School of Medicine, Boston, MA, USA;

^8^VA Boston Healthcare System, Boston, MA and Department of Veterans Affairs Medical Center, Bedford, MA, USA

^10^ Department of Pharmacology, Xiaman Medical College, Xiaman, China;

^11^ Nursing School, Qiqihar Medical University, Qiqihar, Heilongjiang, China;

^12^ Roosevelt University College of Pharmacy, Schaumburg, IL, USA

*Corresponding author:

Wendy Wei Qiao Qiu, M.D., Ph.D.

E-mail: [wqiu67@bu.edu](mailto:wqiu67@bu.edu)

Department of Psychiatry

Department of Pharmacology & Experimental Therapeutics

Boston University School of Medicine

72 East Concord Street, R-623

Boston, MA 02118

Tel: 617-358-1886

Fax: 617-638-5254

**Supplementary Figure 1**


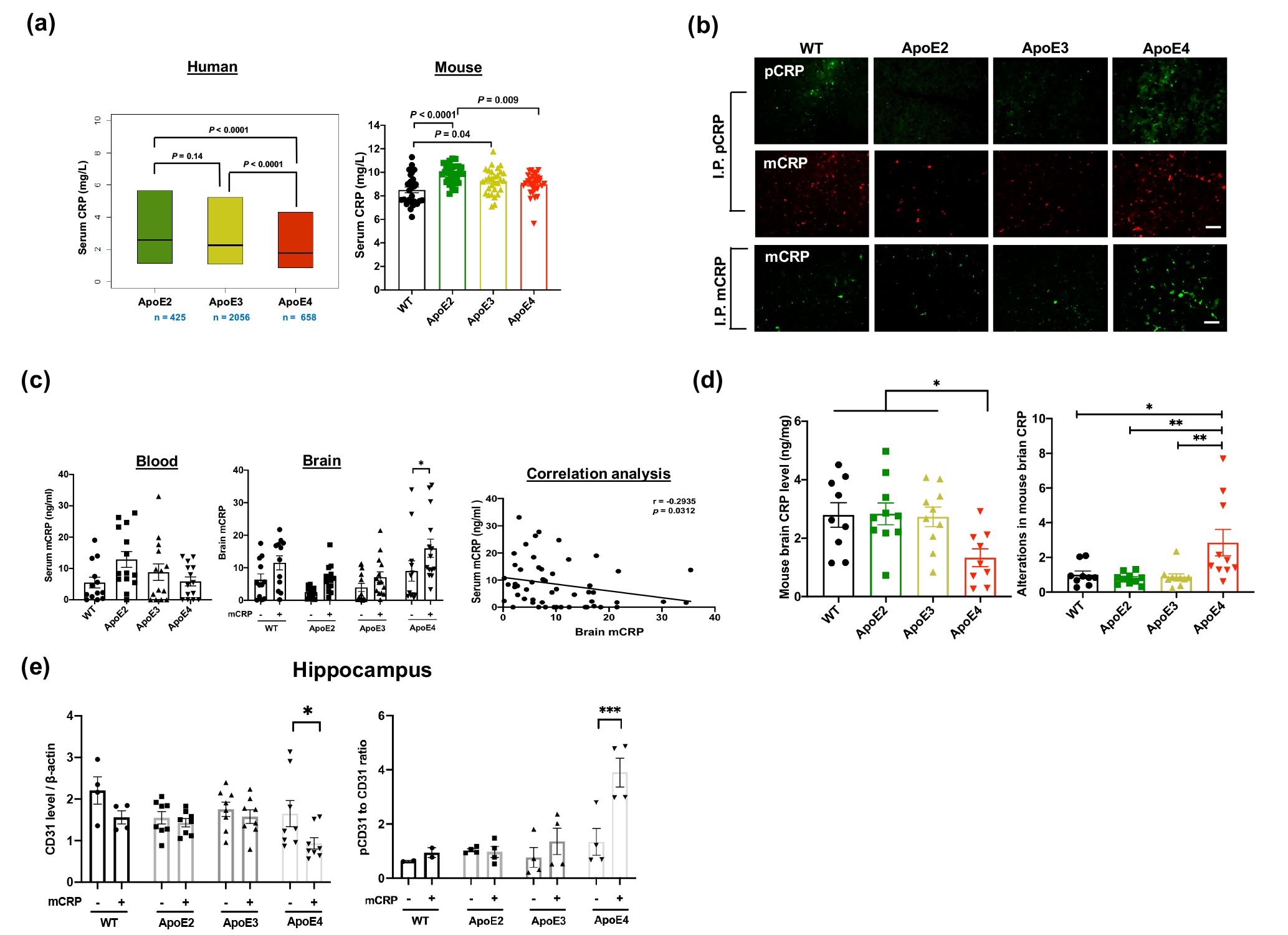


**Supplementary Figure 2**


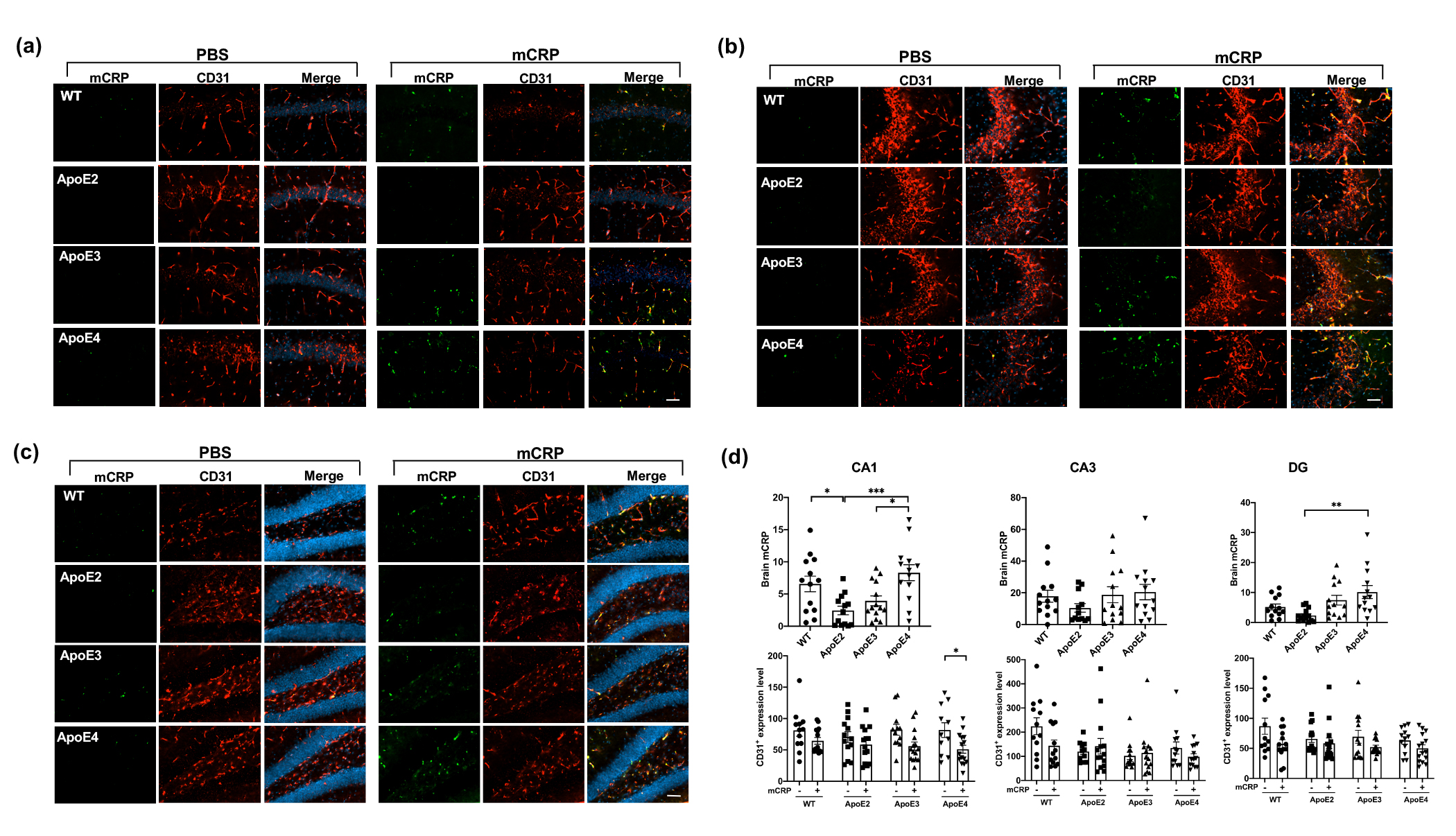


**Supplementary Figure 3**

**
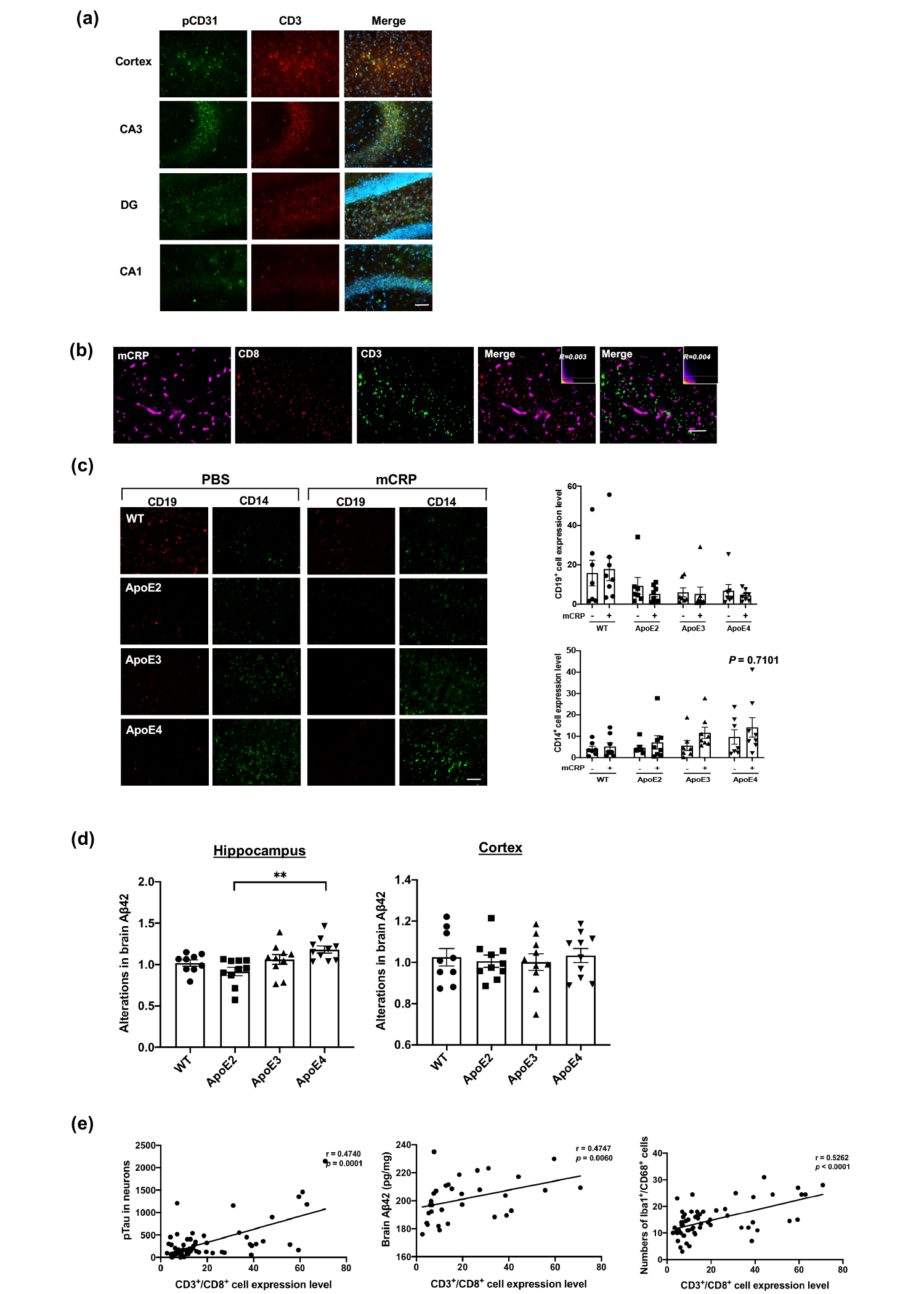
**

**Supplementary Figure 4**

**
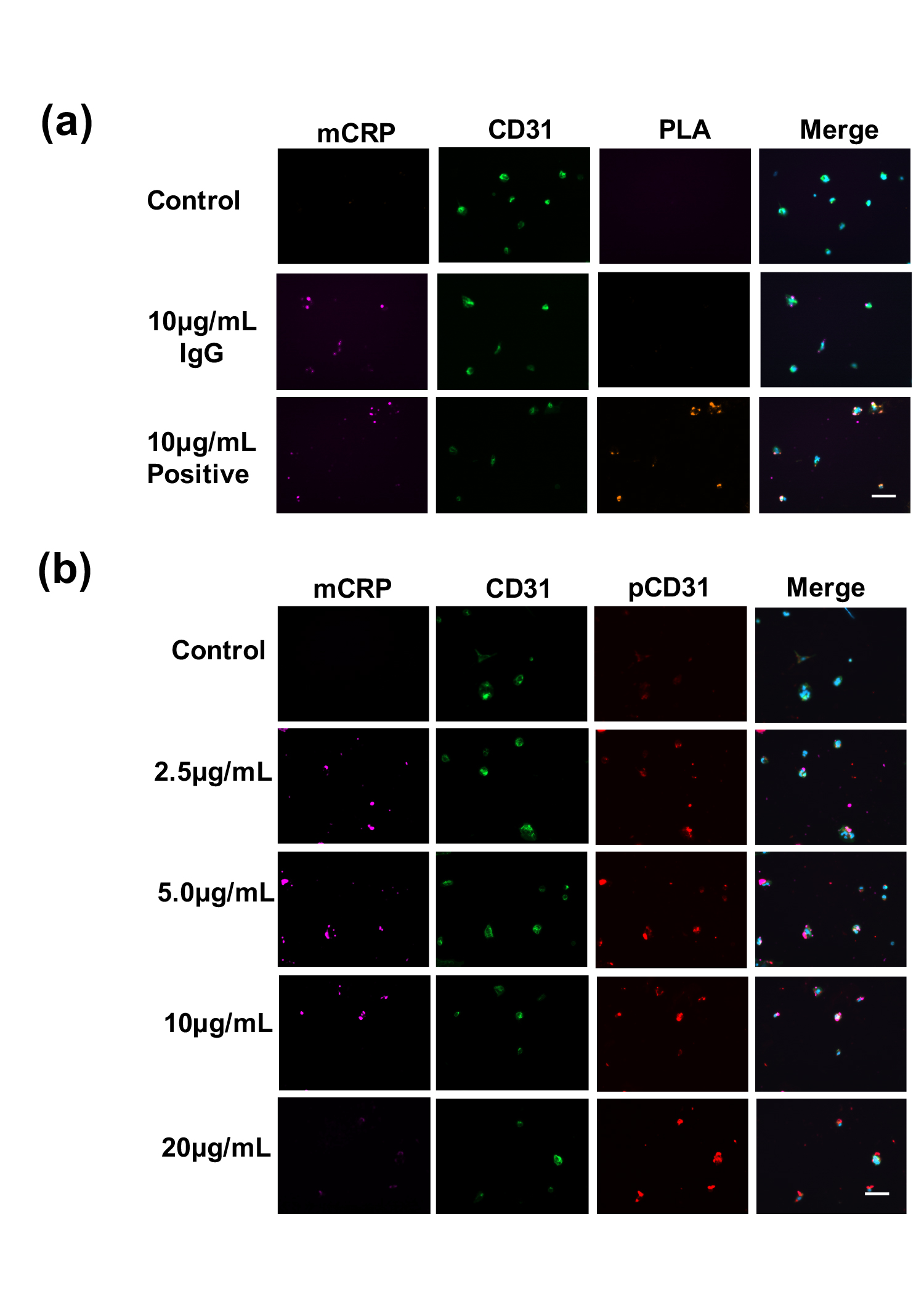
**

**Supplementary Figure 5**

**
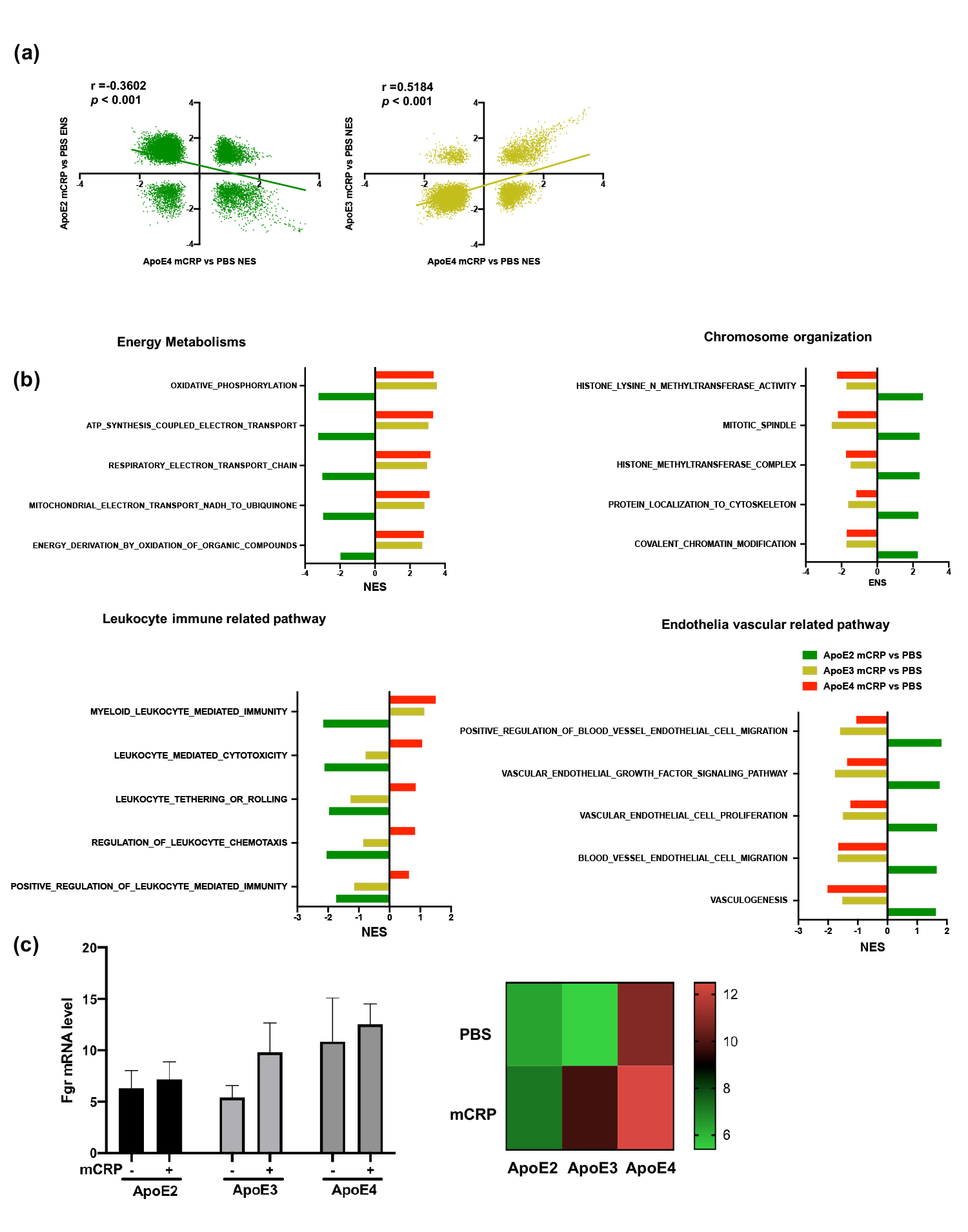
**

**Supplementary Figure 6**

**
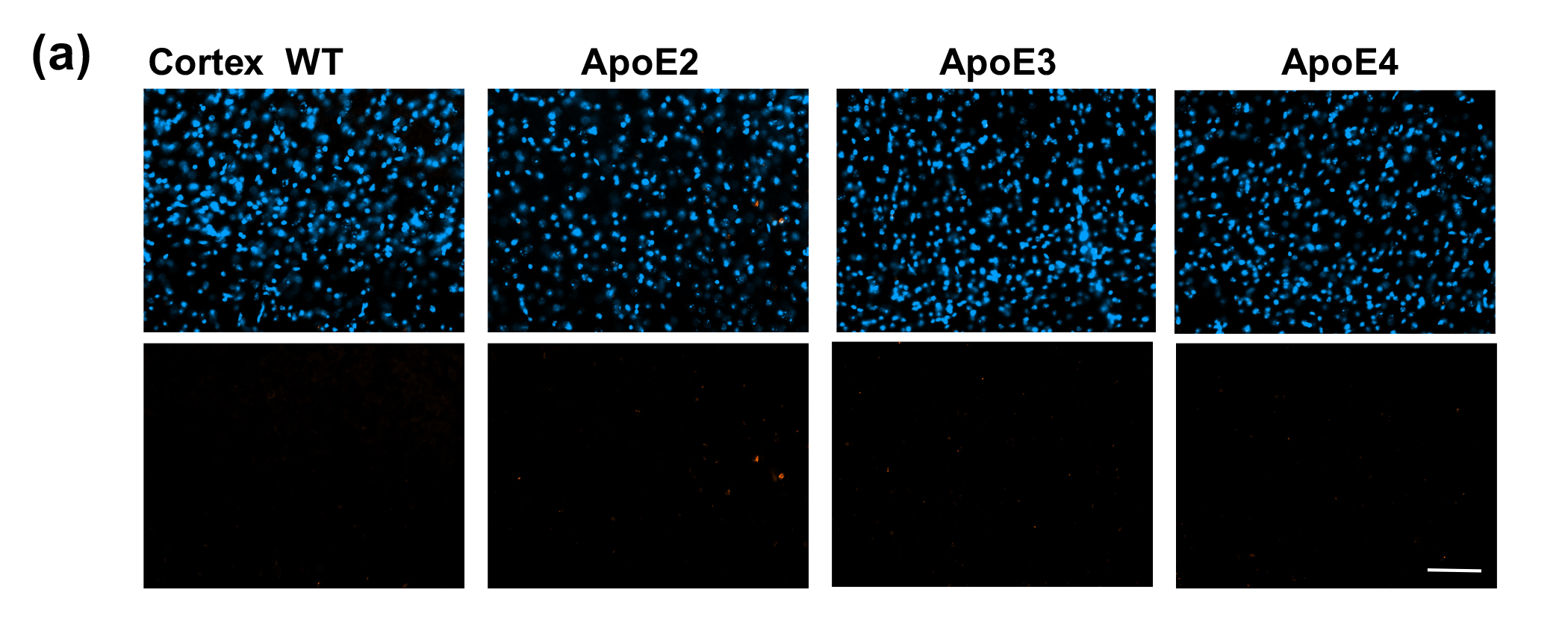
**

**Supplementary Figure 7**


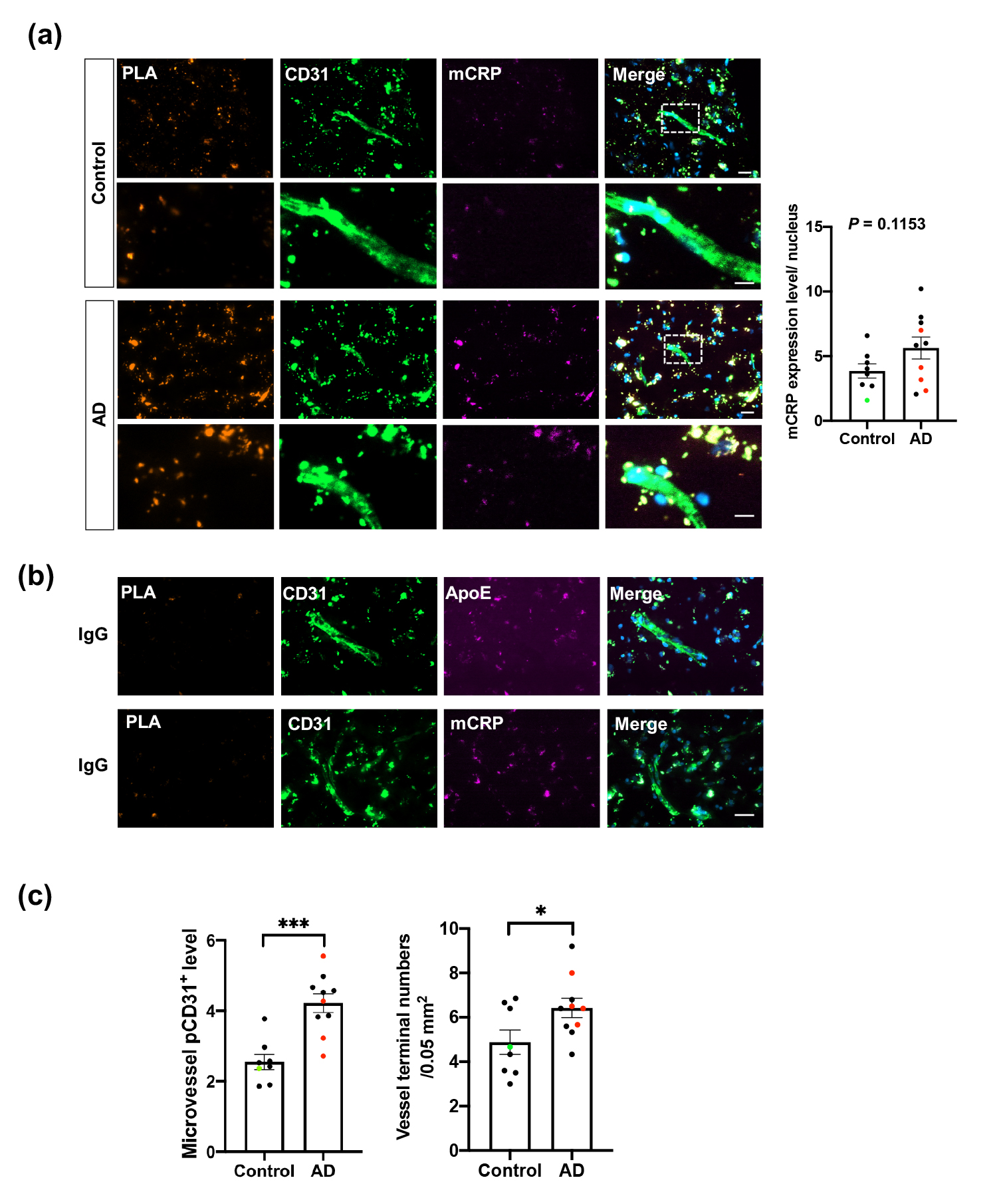

Supplement: Supplementary file 1 — Fig S1‐S7 [file ACEL-20-e13501-s001.docx]
