# Supplementary figures and images for: Monomeric C‐reactive protein via endothelial CD31 for neurovascular inflammation in an ApoE genotype‐dependent pattern: A risk factor for Alzheimer’s disease?
Source: Aging Cell. 2021 Oct 23;20(11):e13501. doi: 10.1111/acel.13501 (PMC8590103; doi:10.1111/acel.13501)

**
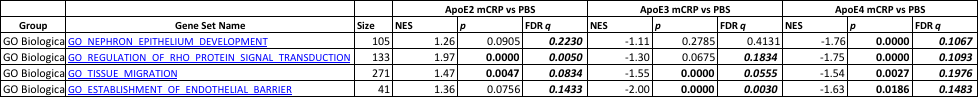
Supplemental Figure 5e**

Supplement: Supplementary file 2 — Fig S5E [file ACEL-20-e13501-s002.docx]
