# Supplementary material for: Monomeric C‐reactive protein via endothelial CD31 for neurovascular inflammation in an ApoE genotype‐dependent pattern: A risk factor for Alzheimer’s disease?
Source: Aging Cell. 2021 Oct 23;20(11):e13501. doi: 10.1111/acel.13501 (PMC8590103; doi:10.1111/acel.13501)
Supplement: Supplementary file 3 — Table S1 [file ACEL-20-e13501-s003.docx]

|  |  | | **Supplement Table 1 Human brain information** | | | | | |  |  |
| --- | --- | --- | --- | --- | --- | --- | --- | --- | --- | --- |
| ID number | | Sex | | Age | Braak Stage | CAA | *APOE* genotype | last time MMSE |  |  |
| #1AD | | 2 | | 92 | 5 | 1 | 3/3 | 15 |  |  |
| #2AD | | 1 | | 79 | 6 | 2 | 3/4 | 11 |  |  |
| #3AD | | 1 | | 76 | 6 | 2 | 3/4 | 13 |  |  |
| #4AD | | 2 | | 88 | 6 | 2 | N/A | N/A |  |  |
| #5AD | | 1 | | 86 | 6 | 2 | 3/3 | 11 |  |  |
| #6AD | | 2 | | 83 | 6 | 3 | 3/3 | 18 |  |  |
| #7AD | | 2 | | 94 | 5 | 3 | N/A | 14 |  |  |
| #8AD | | 1 | | 68 | 6 | 3 | 3/4 | 16 |  |  |
| #9AD | | 1 | | 78 | 5 | 1 | 3/3 | 11 |  |  |
| #10AD | | 1 | | 70 | 6 | 1 | 4/4 | 4 |  |  |
| #11CON | | 1 | | 88 | 4 | 0 | 3/3 | 21 |  |  |
| #12CON | | 2 | | 95 | 2 | 0 | N/A | N/A |  |  |
| #13CON | | 1 | | 87 | 3 | 1 | 3/3 | 29 |  |  |
| #14CON | | 1 | | 84 | 2 | 0 | 3/3 | 30 |  |  |
| #15CON | | 1 | | 96 | 3 | 1 | 2/3 | 27 |  |  |
| #16CON | | 1 | | 92 | 2 | 0 | 3/3 | 30 |  |  |
| #17CON | | 2 | | 73 | 0 | 0 | 3/3 | 26 |  |  |
| #18CON | | 2 | | 85 | 2 | 1 | 3/3 | 30 |  |  |
